# Metagenomic identification of severe pneumonia pathogens in mechanically-ventilated patients: a feasibility and clinical validity study

**DOI:** 10.1186/s12931-019-1218-4

**Published:** 2019-11-27

**Authors:** Libing Yang, Ghady Haidar, Haris Zia, Rachel Nettles, Shulin Qin, Xiaohong Wang, Faraaz Shah, Sarah F. Rapport, Themoula Charalampous, Barbara Methé, Adam Fitch, Alison Morris, Bryan J. McVerry, Justin O’Grady, Georgios D. Kitsios

**Affiliations:** 1 Center for Medicine and the Microbiome, University of Pittsburgh, Pittsburgh, PA USA; 2 Division of Pulmonary, Allergy and Critical Care Medicine, Department of Medicine, University of Pittsburgh School of Medicine and University of Pittsburgh Medical Center, UPMC Montefiore Hospital, NW628, 3459 Fifth Avenue, Pittsburgh, PA 15213 USA; 3 Division of Infectious Diseases, University of Pittsburgh Medical Center and University of Pittsburgh School of Medicine, Pittsburgh, PA USA; 4 Internal Medicine Residency Program, University of Pittsburgh Medical Center McKeesport, McKeesport, PA USA; 5 Veterans Affairs Pittsburgh Healthcare System, Pittsburgh, PA USA; 6 Bob Champion Research and Educational Building, University of East Anglia, Norwich Research Park, Norwich, UK; 7 Department of Immunology, University of Pittsburgh School of Medicine, Pittsburgh, USA; 8 Quadram Institute Bioscience and University of East Anglia, Norwich, UK

**Keywords:** Nanopore, Metagenomics sequencing, Pneumonia, Pathogen detection, Mechanical ventilation

## Abstract

**Background:**

Metagenomic sequencing of respiratory microbial communities for pathogen identification in pneumonia may help overcome the limitations of culture-based methods. We examined the feasibility and clinical validity of rapid-turnaround metagenomics with Nanopore™ sequencing of clinical respiratory specimens.

**Methods:**

We conducted a case-control study of mechanically-ventilated patients with pneumonia (nine culture-positive and five culture-negative) and without pneumonia (eight controls). We collected endotracheal aspirates and applied a microbial DNA enrichment method prior to metagenomic sequencing with the Oxford Nanopore MinION device. For reference, we compared Nanopore results against clinical microbiologic cultures and bacterial 16S rRNA gene sequencing.

**Results:**

Human DNA depletion enabled in depth sequencing of microbial communities. In culture-positive cases, Nanopore revealed communities with high abundance of the bacterial or fungal species isolated by cultures. In four cases with resistant clinical isolates, Nanopore detected antibiotic resistance genes corresponding to the phenotypic resistance in antibiograms. In culture-negative pneumonia, Nanopore revealed probable bacterial pathogens in 1/5 cases and *Candida* colonization in 3/5 cases. In controls, Nanopore showed high abundance of oral bacteria in 5/8 subjects, and identified colonizing respiratory pathogens in other subjects. Nanopore and 16S sequencing showed excellent concordance for the most abundant bacterial taxa.

**Conclusions:**

We demonstrated technical feasibility and proof-of-concept clinical validity of Nanopore metagenomics for severe pneumonia diagnosis, with striking concordance with positive microbiologic cultures, and clinically actionable information obtained from sequencing in culture-negative samples. Prospective studies with real-time metagenomics are warranted to examine the impact on antimicrobial decision-making and clinical outcomes.

## Background

Pneumonia is a primary cause of morbidity and mortality among adults, leading to more than one million hospitalizations every year and high rates of intensive care unit (ICU) admission in the US [1]. The mainstay of pneumonia management is early and appropriate antimicrobial therapy targeting the causative pathogens, balanced with preventing antibiotic overuse and emergence of resistance [2]. Thus, timely and accurate identification of causal pathogens is imperative yet remains challenging due to reliance on culture-based methods with low sensitivity and long turnaround times (48~72 h to actionable results [3]). Recently developed rapid polymerase-chain reaction (PCR) tests represent a significant advancement in the field [4], but these tests can only detect the presence/absence of selected panels of pathogens, and thus are not comprehensive enough in breadth or resolution. Culture-independent methods using next-generation sequencing of microbial communities may help overcome the limitations of current diagnostic testing [5–7].

Our group and others have provided proof-of-concept evidence that sequencing of the bacterial 16S rRNA gene (16S sequencing) in clinical respiratory specimens can provide diagnostic insights beyond standard microbiologic cultures [5, 8–10]. Nevertheless, standard 16S sequencing is not clinically applicable due to limited resolution (providing only genus-level bacterial identification) and lengthy sample processing, library preparation and data acquisition timelines [11]. The advent of Nanopore metagenomic sequencing (Oxford Nanopore Technologies [ONT], UK) has offered unprecedented capacities for real-time, detailed profiling of microbial communities at species level (including viruses and fungi) [12–15]. With recent technical improvements to overcome the high amounts of contaminating human DNA in clinical respiratory samples [16], Nanopore metagenomics may allow for the development of a novel diagnostic approach for pneumonia.

In this proof-of-concept study, we sought to evaluate the technical feasibility and clinical validity of Nanopore metagenomic sequencing for etiologic diagnosis of severe pneumonia in mechanically-ventilated patients in the ICU. Some of the results of these studies have been previously reported in the form of abstracts [17, 18].

## Methods

Detailed methods are provided in the Additional file 1.

### Study design and participants

From June 2018 – March 2019, we carried out a nested case-control feasibility study from an ongoing registry enrolling mechanically-ventilated adult patients with acute respiratory failure in the Medical Intensive Care Unit (MICU) at the University of Pittsburgh Medical Center (UPMC) [5, 19]. Exclusion criteria included inability to obtain informed consent, presence of tracheostomy, or mechanical ventilation for more than 72 h prior to enrollment.

Given the proof-of-concept nature of our study, we aimed to identify cases and controls that would allow for a meaningful comparison contrast of sequencing outputs. Thus, we reviewed enrolled subjects during this recruitment period to identify pneumonia cases with diagnostic certainty on etiologic pathogen diagnosis (based on clinically-available culture methods), probable pneumonia cases with diagnostic uncertainty on the causative pathogen (culture-negative cases) and then, uninfected controls with low or no suspicion of infection. Selection of cases was done prior (and thus without knowledge) of DNA sequencing or host-response biomarker experiments. We diagnosed clinical pneumonia based on consensus committee review of clinical, radiographic, and microbiologic data per established criteria [20]. Through the selection process described above, we identified 14 subjects with a clinical diagnosis of pneumonia: nine with culture-confirmed diagnosis (culture-positive pneumonia group) and five with negative cultures (culture-negative pneumonia group). Culture-positivity was deemed when a probable respiratory pathogen was isolated in clinical microbiologic cultures of respiratory specimens obtained at the discretion of treating physicians (endotracheal aspirate [ETA] in 7 cases, and bronchoalveolar lavage fluid [BALF] in 2 cases [case 2, case 3]). In culture-positive cases, antibiotic susceptibility testing was done as per standard practice at UPMC’s clinical microbiology laboratory, and results were interpreted based on Clinical and Laboratory Standards Institute criteria [21]. We also included eight subjects (controls) who did not have evidence of lower respiratory tract infection and were intubated either for airway protection (*n* = 5) or respiratory failure from cardiogenic pulmonary edema (*n* = 3).

From enrolled subjects, we collected ETAs per our research protocol within the first 48 h from intubation (baseline samples) and then on day 5 post-intubation if the patient remained on mechanical ventilation in the ICU [19, 22]. For the purposes of comparing ETA sequencing results with clinical microbiologic cultures, we utilized baseline samples in 20/22 subjects, because baseline samples were closer to the timing of clinical microbiologic specimen acquisition for diagnosis of index pneumonia (not ventilator-associated pneumonia [VAP]). In two subjects, we utilized ETAs obtained on the fifth day post-intubation (instead of their baseline samples) because in one case (case 10, Additional file 1: Figure S1) the day 5 sample was obtained closer to the timing of clinical cultures for clinical suspicion of VAP, whereas in case 15 the day 5 sample was obtained at a time point that earlier clinical suspicion of pneumonia was refuted and antibiotics were stopped (congestive heart failure control). We utilized only a single specimen per patient in order to maintain our diagnostic inference analyses on patient-level (and not sample-level). From plasma samples taken at the same time with ETAs, we measured plasma procalcitonin levels [19]. We recorded demographic, physiological, and laboratory variables at the time of sample acquisition, from which we calculated clinical pulmonary infection scores (CPIS) [23], and reviewed the antimicrobial therapies administered for the first 10 days from intubation.

This study was approved by the University of Pittsburgh Institutional Review Board (Protocol PRO10110387). Written informed consent was provided by all participants or their surrogates in accordance with the Declaration of Helsinki.

### Microbial DNA sequencing approaches

We focused our sequencing approach on DNA-based organisms (i.e. excluding RNA viruses) and aimed to perform agnostic profiling for microbes (bacteria and fungi) present in the ETAs obtained from the patients in the ICU. We performed direct-from-sample sequencing and we did not use isolated organisms from clinical cultures for sequencing. However, metagenomic microbial DNA sequencing in clinical respiratory samples is technically challenging because of the high amounts of contaminating human DNA that can overwhelm the sequencing output (ratio of human:microbial DNA > 99:1 [24]. Therefore, we applied a human DNA depletion step in ETA samples that utilized a detergent-based (saponin) method for selective lysis of human cells and digestion of human DNA with nuclease, as recently described [16]. We extracted genomic DNA with the DNeasy Powersoil Kit (Qiagen, Germantown, MD) and assessed the efficiency of human DNA depletion by comparing quantitative PCR (qPCR) cycle threshold (Ct) of a human gene (Glyceraldehyde 3-phosphate dehydrogenase - GAPDH) and the bacterial 16S rRNA gene (V3-V4 region) [25] between samples subjected to depletion vs. not (depleted vs. undepleted samples).

From extracted DNA in depleted samples, we prepared metagenomic sequencing libraries with a Rapid PCR Barcoding Kit (SQK-RPB004) and then ran on the MinION device [Oxford Nanopore Technologies (ONT), UK)] for 5 h. We basecalled the output (i.e. converted the sequencing device output into nucleic acid base sequences) with the Guppy software and used the ONT platform, EPI2ME, for quality control, species identification [What’s In My Pot (WIMP) pipeline] and antimicrobial resistance gene analyses [ARMA workflow]. Samples that generated fewer than 300 high-quality microbial reads were excluded from further analyses. As an internal quality control for the reliability and reproducibility of Nanopore sequencing, we performed sequencing on two samples with extracted DNA from a mock microbial community with known composition (ZymoBIOMICS Microbial Community Standard) and compared derived vs. expected abundance of microbial species.

To further validate the results of Nanopore sequencing for bacterial DNA, we performed standard 16S rRNA gene (V4 region) PCR amplification and sequencing on the Illumina MiSeq Platform as a reference method for bacterial DNA sequencing [26]. We processed 16S sequences using an in-house pipeline developed by the University of Pittsburgh Center for Medicine and the Microbiome (CMM) [22, 27–31]. Samples that generated fewer than 100 bacterial reads were excluded from further analyses.

### Ecological and statistical analyses

From sequencing reads obtained from Nanopore and 16S sequencing, we calculated alpha diversity by Shannon index, performed permutational multivariate analysis of variance (PERMANOVA) testing to assess compositional differences between sample types, and visualized compositional dissimilarities between samples with the non-metric multidimensional scaling (NMDS) method using the Bray-Curtis index. All analyses were performed with the R *vegan* package [32].

## Results

### Cohort description

We enrolled 22 mechanical-ventilated patients with acute respiratory failure: nine with retrospective consensus diagnosis of culture-positive pneumonia, five with culture-negative pneumonia, and eight controls. Clinical characteristics and outcomes for the three groups are shown in Table 1. Cases with culture-positive pneumonia had significantly higher CPIS and a trend for higher procalcitonin levels compared to controls (Table 1, Additional file 1: Figure S2). At the time of enrollment, empiric antibiotics had been prescribed for all 14 patients with clinical diagnosis of pneumonia, as well as for 5/8 of control patients (Table 1, Additional file 1: Figure S1).

**Table 1:** Characteristics of enrolled patients. Continuous variables are presented as medians (with interquartile ranges), and categorical variables are presented as N (%)

	Culture-Positive Pneumonia	Culture-Negative Pneumonia	Controls
N	9	5	8
Age, median [IQR], yrs	58.3 [55.2, 62.6]	55.8 [45.7, 62.8]	61.2 [51.9, 67.4]
Male, N (%)	5 (55.6)	2 (40.0)	6 (75.0)
BMI, median [IQR]	24.0 [21.5, 34.6]	31.2 [25.6, 32.9]	28.1 [25.1, 36.1]
SOFA Score, median [IQR]^a^	6.0 [4.0, 6.0]	7.0 [6.0, 7.0]	5.0 [4.0, 8.0]
PaO2:FIO2 ratio, median [IQR], mmHg	158.0 [137.0, 275.0]	150.0 [121.0, 208.0]	221.5 [205.0, 319.5]
Heart rate (median [IQR]), beats per minute	107.0 [92.0, 117.0]	83.0 [82.0, 88.0]	81.5 [73.8, 85.5]
SBP (median [IQR]) mmHg	125.0 [102.0, 141.0]	118.0 [117.0, 127.0]	105.0 [97.8, 117.5]
WBC, median [IQR], × 10^9^ per liter (L)	10.0 [7.4, 16.8]	4.6 [3.5, 8.3]	5.4 [4.5, 11.6]
Platelets, median [IQR], × 10^9^ per liter (L)	190.0 [169.0, 281.0]	155.0 [136.0, 210.0]	141.0 [72.2, 171.0]
Creatinine, median [IQR], mg/dL	1.3 [1.1, 1.5]	1.5 [1.5, 2.0]	1.0 [0.8, 1.4]
Respiratory Rate, median [IQR], 1/min	22.0 [21.0, 24.0]	21.0 [20.0, 24.0]	17.0 [15.5, 17.2]
PEEP, median [IQR], cm	8.0 [5.0, 8.0]	5.0 [5.0, 8.0]	5.0 [5.0, 5.8]
Tidal Volume (per kg of PBW), (median [IQR]), ml/kg	6.8 [6.2, 8.4]	6.2 [6.1, 6.6]	6.3 [6.0, 7.1]
Plateau Pressure, median [IQR], cm	20.0 [13.0, 23.5]	25.0 [21.0, 29.0]	16.0 [13.0, 21.5]
Type of mechanical breaths, n (%)^b^			
Volume control	7 (77.8)	4 (80.0)	8 (100)
Pressure control	2 (22.2)	1 (20.0)	0 (0)
Ventilator free days, median [IQR], days	12.0 [0.0, 23.0]	17.0 [6.0, 23.0]	24.5 [24.0, 26.0]
ICU Length of Stay, median [IQR], days	8.0 [5.0, 18.0]	11.0 [6.0, 12.0]	4.5 [3.8, 5.0]
Acute Kidney Injury, N (%)	8 (88.9)	4 (80.0)	2 (25.0)
30 Day Mortality, N (%)	3 (33.3)	1 (20.0)	1 (12.5)
On antibiotic therapy, N (%)	9 (100)	5 (100)	5 (62.5)
Antibiotic days, median [IQR], days	12.0 [10.0, 24.0]	21.0 [20.0, 24.0]	3.5 [0.0, 7.5]
CPIS, median [IQR],	8.0 [7.0, 9.0]	6.0 [5.0, 7.0]	5.0 [4.0, 5.2]
Procalcitonin, median [IQR], pg/μl	2783.0 [1049.5, 4330.1]	4866.0 [94.4, 4965.1]	353.5 [250.4, 1531.6]

*Abbreviations*: *IQR* Interquartile range, *BMI* Body mass index, *SOFA* Sequential organ failure assessment, *PaO*_*2*_ Partial pressure of arterial oxygen, *FiO*_*2*_ Fractional inhaled concentration of oxygen, *SBP* Systolic blood pressure, *WBC* White blood cell count, *PEEP* Positive end-expiratory pressure, *PBW* Predicted body weight, *ICU* Intensive care unit, *CPIS* Clinical pulmonary infection score

^a^ SOFA score calculation does not include the neurologic component of SOFA score because all patients were intubated and receiving sedative medications, impairing our ability to perform assessment of the Glasgow Coma Scale in a consistent and reproducible fashion

^b^ All patients were on assist controls mode of ventilation at time of sample acquisition

### Technical feasibility of Nanopore sequencing in clinical samples

**Fig. 1:**
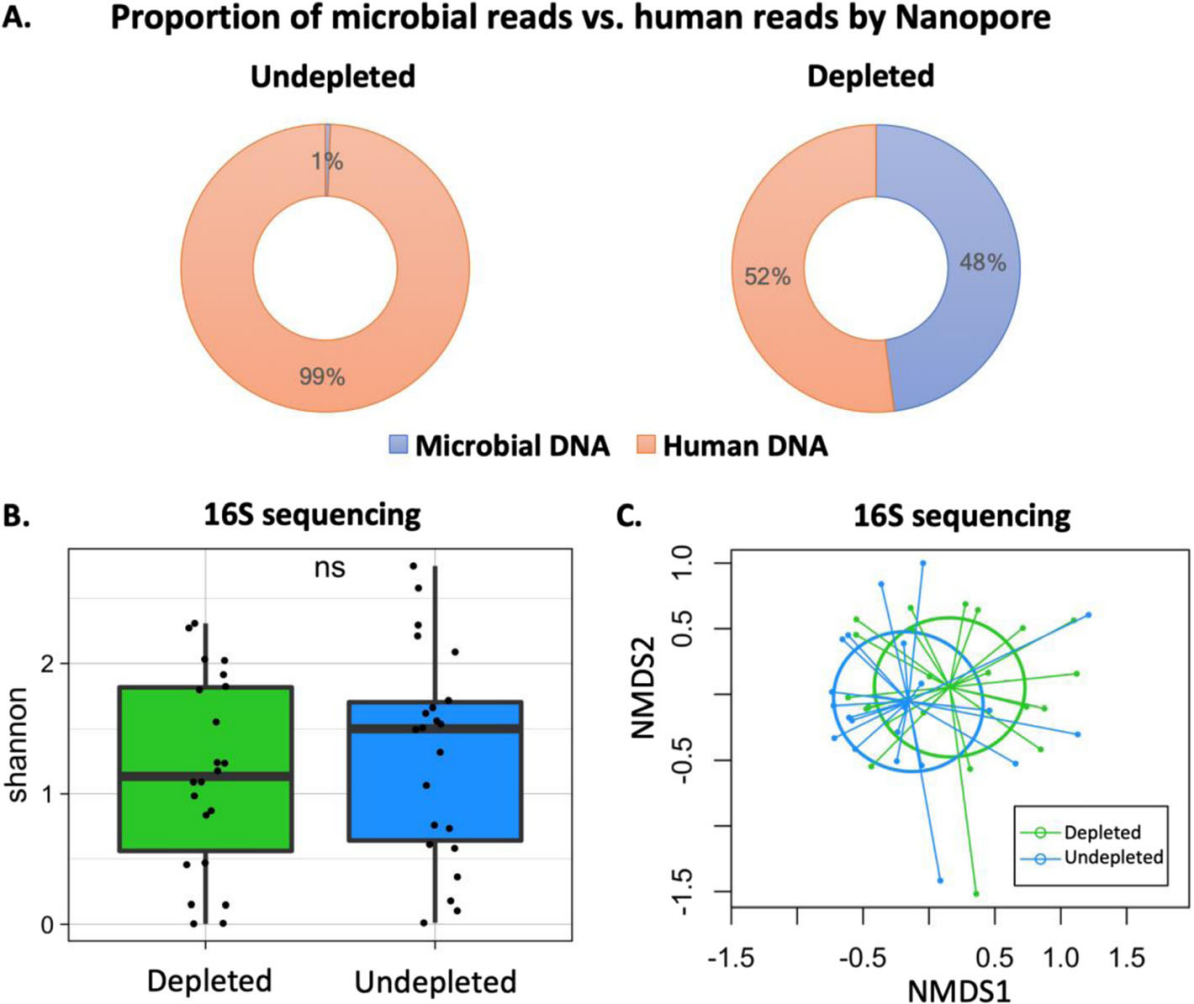
Saponin-based human DNA depletion effectively removed human DNA without changing bacterial community structure. **a** Before human DNA depletion, 1% of Nanopore reads were of microbial origin; following human DNA depletion, 48% of Nanopore reads were of Microbial origin. **b** There was no significant difference in alpha diversity of bacterial communities between depleted and undepleted samples assessed by 16S rRNA gene sequencing. **c** Non-metric multidimensional scaling (NMDS) plot of the Bray-Curtis dissimilarity index between depleted and undepleted samples based on 16S rRNA gene sequencing. Depleted samples were compositionally similar to undepleted samples (PERMANOVA, *p*-value = 0.17)

Pre-processing of the ETA samples with the saponin-based human DNA depletion protocol resulted in relative enrichment of bacterial DNA by an average of 1260-fold (Additional file 1: Figure S3). This microbial enrichment step allowed for generation of sufficient numbers of microbial reads by Nanopore sequencing in depleted samples (median 6682 reads, average proportion 48% of total reads), whereas in undepleted samples the sequencing output was overwhelmed by human DNA (only 1% of reads were of microbial origin) and effectively was unusable (Fig. 1a). Although this enrichment step allowed for generation of analyzable sequencing output in depleted samples (i.e. 48% of total reads), we sought to define whether the depletion protocol altered the underlying microbial communities in any detectable way. For that reason, we compared 16S rRNA gene sequencing profiles between depleted and undepleted samples. We elected to perform 16S-based comparisons because amplicon-based sequencing methods are unaffected by the amount of contaminating human DNA. Importantly, depleted and undepleted samples by 16S rRNA gene sequencing demonstrated no significant differences in alpha or beta diversity (Fig. 1b, c), suggesting that the underlying microbial community in depleted samples was closely representative of the raw, undepleted samples that were not subjected to any additional processing.

### Analytical validity of Nanopore sequencing

Nanopore-derived bacterial communities showed striking similarity with both mock communities of extracted DNA (Additional file 1: Table S1) as well as 16S-derived community profiles for bacteria from clinical samples (Additional file 1: Figure S1), underscoring the analytical validity of Nanopore results for use in further analyses.

### Nanopore community profiles by clinical group

**Fig. 2:**
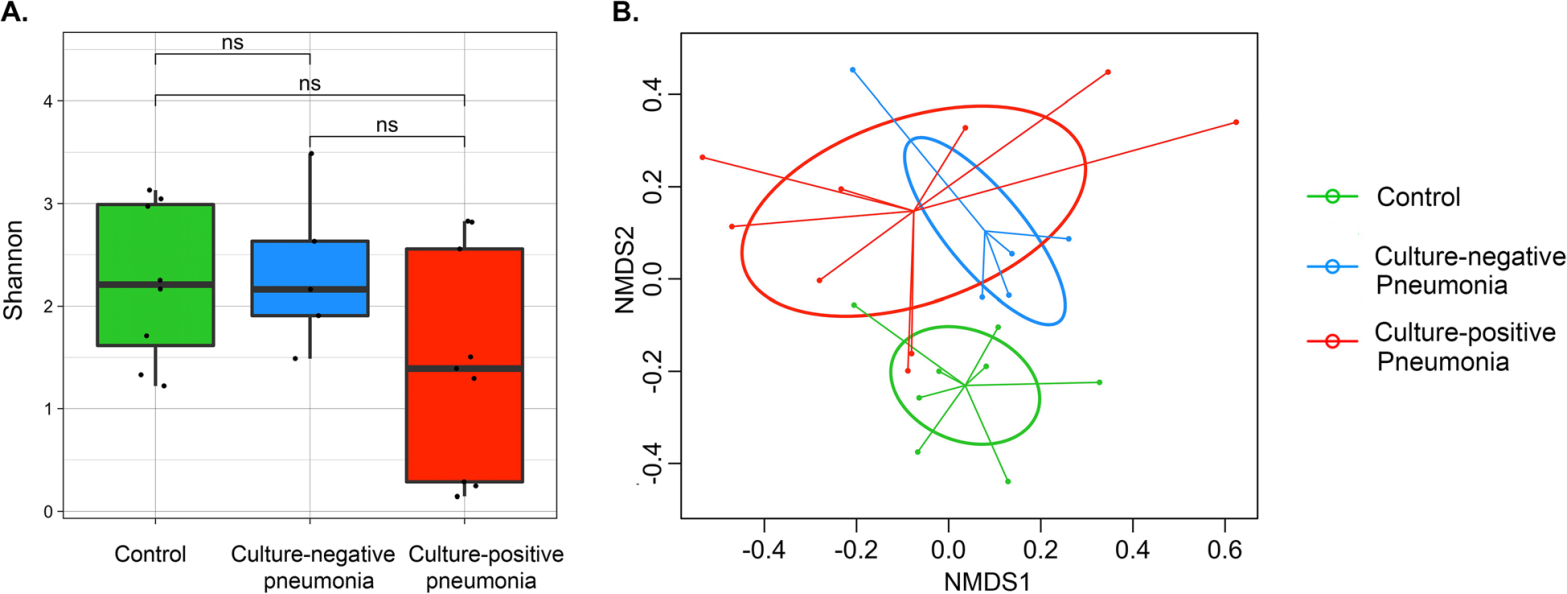
Comparisons of lung microbiome between culture-positive pneumonia, culture-negative pneumonia and controls based on Nanopore sequencing. **a** Compared to samples from patients with culture-negative pneumonia, culture-positive samples had a trend for lower alpha diversity of lung microbial communities by Shannon index. **b** By non-metric multidimensional scaling (NMDS) plot of the Bray-Curtis dissimilarity index, there were significant differences in overall microbial community compositions between three groups (PERMANOVA for Bray-Curtis dissimilarity index, *p* value= 0.038, R^2^ = 0.120)

By Nanopore sequencing, culture-positive samples had a trend for lower alpha diversity (Shannon index) compared to culture-negative samples (Fig. 2a and Additional file 1: Figure S4A) and demonstrated global compositional dissimilarities compared to culture-negative and control samples (PERMANOVA *p*-value = 0.038, R^2^ = 0.12, Fig. 2b and Additional file 1: Figure S4B).

### Nanopore-based pathogen identification

#### Culture-positive pneumonia

We first examined Nanopore results in the culture-positive cases in which microbiologic confirmation of the causative pathogen allowed for a targeted interrogation of the sequencing output for the corresponding microbial species. We examined different thresholds of sequencing output (i.e. absolute number of reads for the dominant pathogen vs. relative or ranked abundance thresholds for pathogens) to maximize sensitivity of Nanopore results for detecting the culture-identified pathogen(s). By focusing on the three most abundant species (bacterial or fungal) by Nanopore sequencing, we were able to identify all culture-confirmed pathogens with high relative abundances.

**Fig. 3:**
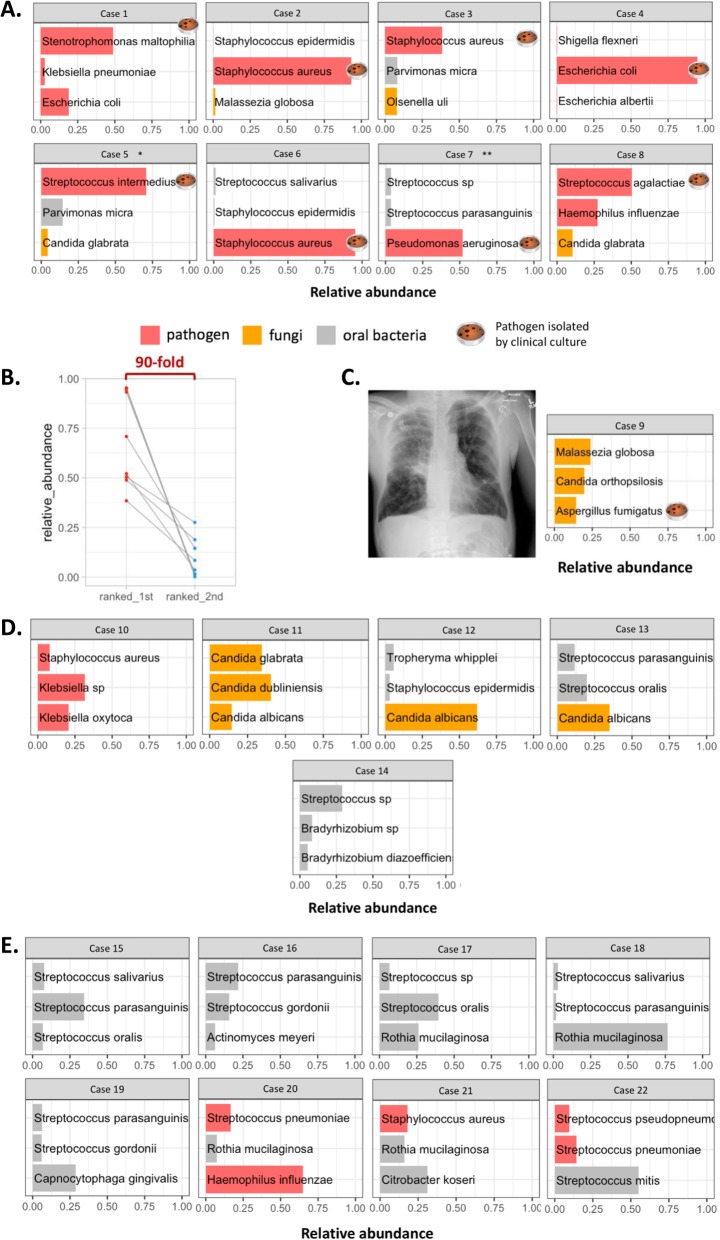
Comparisons of microbes detected by Nanopore metagenomic sequencing and clinical culture. Each small plot represents an endotracheal aspirate; each bar represents a microbe; the X-axis represents the relative abundance of microbes by Nanopore. Petri dish represents pathogen isolated by clinical culture. The three most abundant taxa detected by Nanopore sequencing were included. **a** In 8 samples with culture-positive bacterial pneumonia, Nanopore signals were dominated by pathogens isolated by culture. **b** In 8 samples with culture-positive bacterial pneumonia, the relative abundance of culture-positive pathogens was 90-times higher than that of the second-ranked taxa detected by Nanopore. **c** In 1 sample with probable invasive fungal infection, chest radiograph supported a clinical diagnosis of pneumonia, *Aspergillus fumigatus* was isolated by culture, and Nanopore revealed the same fungal pathogen by sequencing. **d** In 5 culture-negative pneumonia samples, potential pathogens were found in one sample, and fungi were found in 3 samples with Nanopore. **e** Only typical oral bacteria were identified in 5/8 of control samples, but potential pathogens were detected in 3/8 of them. * compared to culture of pleural fluid; ** case of culture-positive tracheobronchitis and acute exacerbation of chronic obstructive pulmonary disease (no infiltrate on chest radiograph)

In eight culture-positive bacterial pneumonias, Nanopore profiles showed high abundance of the same bacterial species isolated in cultures (Fig. 3a). These highly abundant causative bacterial pathogens had on average 90 times higher relative abundance compared to the species ranked second in abundance in each community (Fig. 3b). Nanopore sequencing also revealed high abundance of additional potential bacterial pathogens in 2/8 of samples that were not detected by cultures (*E. coli* in subject 1 and *H. influenzae* in subject 8), suggesting the presence of a polymicrobial infection despite the isolation of a single pathogenic bacterial species on standard cultures (Fig. 3a).

These eight culture-positive cases with clinical antibiograms allowed for examination of the potential predictive utility of antibiotic resistance gene detection with metagenomic sequencing (Table 2). In the single case of methicillin-resistant *Staphylococcus aureus* (MRSA, case 2, Additional file 1: Figure S1), Nanopore detected 4 reads aligned to the responsible *mecA* gene, whereas in the three cases of methicillin-sensitive *S. aureus* (MSSA, cases 3, 5 and 6), no *mecA* gene reads were detected. Similarly, in the two cases of *Stenotrophomonas maltophilia* and *E.coli*, Nanopore detected genes that explained the observed phenotypic antimicrobial resistance profile (Table 2).

**Table 2:** Comparison of antibiotic resistance phenotype and clinically relevant resistance genes in cases of bacterial pneumonia. The antibiotic resistance phenotype was detected by clinical culture and clinically relevant resistance genes were detected by Nanopore. Genes conferring resistance phenotype are highlighted in bold

Case	Pathogen by culture & Nanopore	Resistance phenotype by culture	Clinically relevant resistance genes [N of alignments]
Resistance identified			
Case 1	*Stenotrophomonas maltophilia*	R: ticarcillin/clavulanic acid R: ceftazidime	blaTEM-4 [4] blaTEM-112 [1] blaTEM-157 [1] blaACT-5 [1]
		I: levofloxacin	oqxB [1]
		Tetracycline not tested	*tetC* [6]
Case 2	*Staphylococcus aureus*	R: methicillin	mecA [4]
		R: erythromycin, clindamycin	ermA [10] erm (33) [1]
			*tet38* [11] *ant(4′)-lb* [9] *tetC* [3] *blaTEM-4* [1]
Case 3	*Staphylococcus aureus*	I: tetracycline	tetK [1] tet38 [1] tetQ [1]
Case 4	*Escherichia coli*	R: tetracycline	tetX [1]
		R: Trimethoprim-sulfamethoxazole R: ciprofloxacin, levofloxacin	sul1 [363] dfrA [127]
			acrF [315]^a^ parE [304]^a^ mfd [277]^a^
			*mphA* [215] *aadA5* [196] *vgaC* [110] *blaACT-5* [7] *blaACT-14* [3] *mefA* [1] *mel* [1]
No resistance identified			
Case 5	*Staphylococcus aureus*	S: all tested agents	none
Case 6	*Staphylococcus aureus*	S: all tested agents	*tet38* [2] *blaTEM4* [2]
Case 7	*Pseudomonas aeruginosa*	S: all tested agents	none
Resistance not tested			
Case 8	*Streptococcus agalactiae*	Not tested	*tetM* [60] *isaC* [55] *sul1* [2] *tetQ* [2] *mphA* [1] *aadA5* [1]

*Abbreviations*: *R* Resistant, *I* Intermediate, *S* Susceptible

^a^ Genes conferring antibiotic resistance phenotype but not classified as clinical relevant genes by EPI2ME antimicrobial resistance gene analyses [ARMA workflow]

Tested agents for case 5: Ampicillin/Sulbactum, Oxacillin, Imipenem, Gentamicin, Erthromycin, Tetracycline, Vancomycin, Clindamycin, Linezolid, Rifampin, Trimethoprim-sulfamethoxazole, Synercid

Tested agents for case 6: Ampicillin/Sulbactum, Oxacillin, Imipenem, Gentamicin, Erthromycin, Tetracycline

Tested agents for case 7: Piperacillin/Tazobactum, Ticarcillin/Clavulanic acid, Cefepime, Ceftazidime, Imipenem, Meropenem, Aztreonam, Gentamicin, Tobramycin, Amikacin, Ciprofloxacin, Levofloxacin

We also tested the performance of Nanopore sequencing in one case with probable invasive fungal infection. Subject 9 was a lung transplant recipient who had been receiving antifungal therapy for a positive sputum culture for *Aspergillus fumigatus*. Clinical decompensation with acute respiratory failure raised concern for bacterial co-infection and initiation of intensive broad-spectrum antibiotics. BALF culture grew again *Aspergillus fumigatus,* which was the dominant pathogen detected by Nanopore, without any other sequencing evidence of bacterial infection (Fig. 3c). Thus, in this case with probable invasive fungal infection, Nanopore sequencing only identified a confirmed fungal pathogen and ruled out the presence of bacterial pneumonia.

#### Culture-negative pneumonia

Nanopore sequencing provided diverse representations of microbial communities in cases of clinical suspicion of pneumonia with negative cultures, and thus interpretation needs to be individualized for each case (Additional file 1: Figure S1).

In case 10 with an initial diagnosis of aspiration pneumonia caused by *S. aureus* and *Klebsiella oxytoca* identified by BALF culture on day 2 post-intubation, clinical deterioration on appropriate antibiotic therapy and new radiographic infiltrates by day 5 raised concern for VAP. However, repeat BALF culture on day 5 was negative. Nanopore detected high abundance of both *S. aureus* and *Klebsiella oxytoca* on day 5 sample, revealing that the culture-negative community consisted of abundant previously identified pathogens, which were likely not viable at the time of the day 5 BALF acquisition due to ongoing antibiotic therapy. Moreover, low procalcitonin level at the time of the day 5 sample (94 pg/μl) and absence of new pathogens by sequencing made diagnosis of new VAP unlikely.

In another case of a lung transplant recipient (subject 11) with diffuse bilateral consolidations and persistent clinical septic picture of undefined etiology, BALF culture was only positive for yeast and the patient was empirically treated with broad-spectrum antibiotics. Eventually, the patient was proven to be fungemic with delayed growth of *Candida glabrata* on initial blood cultures prompting addition of antifungal therapy. Nanopore sequencing on ETA sample from day 1 post-intubation showed high abundance of *Candida glabrata* and *Candida dubliniensis* with very low abundance of bacterial reads, confirming the absence of bacterial pneumonia and demonstrating fungal colonization of the allograft. Of note, in two other culture-negative cases, Nanopore also detected high abundance of *Candida albicans* (Fig. 3d), whereas in the last case, both Nanopore and 16S sequencing identified abundant oral bacteria with no typical respiratory pathogens.

#### Controls

Eight control subjects did not meet clinical diagnostic criteria for pneumonia on retrospective examination of their clinical course. Despite not meeting diagnostic criteria, 5/8 cases empiric antibiotics were prescribed empiric antibiotics early in their course for initial consideration of possible pneumonia. Five samples were dominated by common oral bacteria, such as *Rothia* and non-*pneumoniae Streptococcus* species [33, 34]. However, in the other three samples, Nanopore and 16S sequencing detected potential respiratory pathogens (e.g. *S.aureus*, *H. influenzae* or *S. pneumoniae*) that were likely airway colonizers not causing clinical infection, notion supported by clinical improvement despite early discontinuation of antibiotics and/or low procalcitonin levels (Additional file 1: Figure S1). No significant fungal DNA presence was detected by Nanopore sequencing in the control group (Fig. 3e).

## Discussion

In this nested case-control study, we provide proof-of-concept evidence that untargeted, shotgun metagenomic sequencing with the MinION device can reveal clinically useful information for etiologic diagnosis of pneumonia in mechanically-ventilated patients. We demonstrate feasibility of metagenomic sequencing directly from clinical respiratory specimens by applying a saponin-based protocol for human DNA depletion prior to sequencing. Our analyses highlighted global microbial community structure and species-level compositional differences associated with culture-positivity and clinical diagnosis of pneumonia. Nanopore sequencing had good concordance with cultures by detecting high abundance of the causative pathogenic bacteria in culture-positive cases, but also revealed profiles with low abundance of typical respiratory pathogens in selected culture-negative cases with clinical suspicion of bacterial pneumonia.

Nanopore metagenomic sequencing holds promise as a potential diagnostic tool due to its comprehensive scope, in-depth resolution with long read sequencing and real-time data generation [6]. However, contaminating human DNA has been a rate-limiting step for clinical implementation. By applying a recently validated protocol with saponin-based, human DNA depletion [16], we demonstrate that this approach is feasible, reproducible and effective for maximizing the microbial signal in clinical samples and providing interpretable sequencing output.

Nanopore sequencing showed high accuracy in pathogen identification in culture-positive pneumonia. The high concordance of our sequencing results with clinically-obtained microbiologic studies, despite the use of different samples (research ETAs vs. clinical ETA or BALF) highlights the robustness of DNA-based sequencing approaches for pathogen identification. Obviating the need for ex-vivo growth for organisms, direct-from-sample sequencing can offer comprehensive snapshots of the component microbes of the communities at the time of sample acquisition. Sequencing methods are thus robust to specific growth condition requirements or the impaired viability of organisms due to antecedent antimicrobials [35]. In exploratory analyses of the sequencing output, Nanopore also provided antibiotic resistance information by detecting clinically relevant resistance genes that matched the phenotypic resistance on antibiograms (e.g. *mecA* detection/absence in MRSA/MSSA cases, respectively). Overall, rapid metagenomic sequencing closely matched the results of current, reference-standard diagnostic methods in our cohort, which typically take 2–3 days for allowing appropriate antibiotic adjustments to occur. Thus, with further external validation in additional cohorts, nanopore metagenomics have the potential to shorten the time to etiologic diagnosis and appropriate antimicrobial therapy selection.

Invasive pulmonary fungal infections represent a major diagnostic challenge due to the poor sensitivity and slow turnaround times of cultures [36]. In the single case of *Aspergillus fumigatus* infection, Nanopore confirmed the high abundance of *Aspergillus fumigatus* in the community and ruled out concomitant bacterial pneumonia. Such results can directly influence clinical practice, as unnecessary and potentially harmful antibiotics could be discontinued, with antimicrobial therapies focused on the causative fungal pathogen [37].

In cases of culture-negative pneumonia and in controls, the main compositional pattern consisted of diverse communities with oral bacteria abundance [5], similar to clinical microbiology reports of normal respiratory flora. In such cases, early de-escalation or discontinuation of antibiotics could be further supported by sequencing results. Nonetheless, in 3/5 airway controls, potential respiratory pathogens were detected in high abundance by both Nanopore and 16S sequencing, in the absence of other supportive evidence of pneumonia. Such cases highlight an important clinical challenge that can emerge from the high sensitivity of sequencing technologies for identifying pathogenic organisms missed by cultures. Detection of abundant DNA from respiratory pathogens in the airways of mechanically-ventilated patients does not translate into a clinical diagnosis of pneumonia or need for antibiotic administration. As the field of sequencing-based diagnostics is nascent, we currently do not have diagnostic thresholds for distinguishing colonizing vs. infecting organisms, similar to the ones considered for culture-based methods based on numbers of colony-forming units [38]. However, the distinction between colonization and infection cannot be based solely on culture results or microbial DNA sequencing outputs, but needs to be an integrative one, incorporating clinical, radiographic, and systemic/focal host-responses [39–41]. With the introduction of RNA-based sequencing, a simultaneous assessment of microbial community profiles with the corresponding host transcriptomics at the local level may offer further diagnostic insights [40]. At the same time, knowledge of colonizing organisms in critically-ill patients can facilitate more targeted choices for initial antibiotic regimens in the event of a secondary infection, such as VAP.

Our study is limited by the single center design and the available sample size. We did not perform Nanopore sequencing and data analyses in real-time because of our retrospective study design, and our objective of demonstrating proof-of-concept feasibility as opposed to practical clinical utility. Nonetheless, the method is implementable with short turnaround times (~ 6-8 h) [13]. The results of antibiotic resistance gene sequencing should be interpreted with caution, and development of reliable predictive rules for pneumonia diagnosis or resistance gene identification based on sequencing outputs will require prospective examination of large cohorts of patient samples. Finally, the human DNA depletion method we applied is not yet optimized for viral DNA detection [16].

In conclusion, our study demonstrates technical feasibility and clinical validity of direct-from-specimen metagenomics with a rapid protocol for human DNA depletion protocol and sequencing with the MiNION device. Metagenomic approaches hold promise for the development of rapid and comprehensive diagnostic tools for severe pneumonia that could transform the existing diagnostic paradigm. With real-time data generation and turnaround times of 6-8 h from sample acquisition to result, rapid metagenomics could conceivably allow for targeted adjustment of initial empiric antibiotic regimens even before their second dose is due, and thus allow for personalized antimicrobial prescriptions and antibiotic stewardship gains. Our results provide rationale for prospective, large-scale studies with real-time application of metagenomics in order to measure the direct impact on antibiotic guidance and clinical outcomes.

## Supplementary information

**Additional file 1: **Expanded Methods. **Figure S1.** Clinical course description and comparisons of microbiologic cultures with sequencing results for all patients enrolled. **Figure S2.** Comparison of clinical pulmonary infection scores (CPIS) and plasma procalcitonin between the three clinical groups. **Figure S3.** Human DNA depletion resulted in reduction of human DNA by 1260-fold without changing bacterial DNA levels. **Table S1.** Nanopore sequencing effectively reproduces the expected composition of a mock microbial community. **Figure S4.** Comparisons of lung microbial communities between culture-positive and culture-negative samples by Nanopore sequencing.

## Data Availability

All de-identified sequencing data were submitted to Sequence Read Archive (SRA) database, accession numbers PRJNA554461. All de-identified datasets for this study are provided in https://github.com/MicrobiomeALIR.

## Abbreviations

ALIR: Acute Lung Injury Registry
BALF: Bronchoalveolar lavage fluid
BMI: Body mass index
CMM: Center for Medicine and the Microbiome
CPIS: Clinical pulmonary infection scores
Ct: Cycle threshold
ETA: Endotracheal aspirate
FiO_2_: Fractional inhaled concentration of oxygen
GAPDH: Glyceraldehyde 3-phosphate dehydrogenase
I: Intermediate
ICU: Intensive care unit
IQR: Interquartile range
MICU: Medical Intensive Care Unit
MRSA: Methicillin-resistant *Staphylococcus aureus*
MSSA: Methicillin-sensitive *S. aureus*
NMDS: Non-metric multidimensional scaling
ONT: Oxford Nanopore Technologies
PaO_2_: Partial pressure of arterial oxygen
PBW: Predicted body weight
PCR: Polymerase-chain reaction
PEEP: Positive end-expiratory pressure
PERMANOVA: Permutational multivariate analysis of variance
qPCR: Quantitative polymerase-chain reaction
R: Resistant
S: Susceptible
SBP: Systolic blood pressure
SOFA: Sequential organ failure assessment
UPMC: University of Pittsburgh Medical Center
VAP: Ventilator-associated pneumonia
WBC: White blood cell count
WIMP: What’s In My Pot
