# Supplementary material for: Metagenomic identification of severe pneumonia pathogens in mechanically-ventilated patients: a feasibility and clinical validity study
Source: Respir Res. 2019 Nov 27;20:265. doi: 10.1186/s12931-019-1218-4 (PMC6882222; doi:10.1186/s12931-019-1218-4)
Supplement: Supplementary file 1 — Additional file 1: Expanded Methods. Figure S1. Clinical course description and comparisons of microbiologic cultures with sequencing results for all patients enrolled. Figure S2. Comparison of clinical pulmonary infection scores (CPIS) and plasma procalcitonin between the three clinical groups. Figure S3. Human DNA depletion resulted in reduction of human DNA by 1260-fold without changing bacterial DNA levels. Table S1. Nanopore sequencing effectively reproduces the expected composition of a mock microbial community. Figure S4. Comparisons of lung microbial communities between culture-positive and culture-negative samples by Nanopore sequencing. [file 12931_2019_1218_MOESM1_ESM.pdf]

Metagenomic Identification of Severe Pneumonia Pathogens in Mechanically-Ventilated Patients: a feasibility and clinical validity study

Supplement

Expanded Methods

Sample collection

Endotracheal Aspirate (ETA): Distal tracheal secretions were suctioned through a closed endotracheal tube suctioning system. In cases that suctioning did not return adequate amount (>5ml) for processing, we instilled 5cc of sterile saline through the tubing system and then repeated suctioning as typically done during the collection of ETA for clinical microbiologic studies at our institution. Samples were directly collected in sputum collection traps and stored at 4°C for up to 72 h before processing. Storage at 4°C (instead of deep freezing to -80°C) was necessary to allow for the completion of the human DNA depletion protocol (described below), which relies on the presence of human and bacterial cells with intact cell membranes.

Plasma samples: Blood samples were centrifuged for separation of plasma at 800g at 4°C for 10min, and plasma was then frozen and stored at -80°C.

Human DNA depletion and bacterial DNA extraction

Sputasol (Oxoid, Hampshire, England) was used to liquefy ETA samples (400 µl) at 1:1 mix. For quality controls, 400 µl of sputasol-treated ETA was further processed for human DNA depletion (referred as "depleted"), while 400 µl of treated ETA was not subjected to human DNA depletion (referred as "undepleted"). We used 2.5% saponin (Sigma, St.Louis, MO) to promote human cell lysis and then added HL-

SAN DNase (ArcticZymes, Tromso, Norway) to remove human DNA according to a recently reported methodology (1). We extracted DNA from both depleted and undepleted ETA samples using DNeasy Powersoil Kits (Qiagen, Germantown, MD), following the manufacturer's instructions.

Nanopore metagenomic sequencing and analyses

We performed microbial metagenomics sequencing using MinION (Oxford Nanopore Technologies (ONT), UK). Five samples plus one DNA extraction negative control using different barcodes were sequenced in each run. Sequencing library was prepared according to the manufacturer's instruction of Rapid PCR Barcoding Kit (SQK-RPB004, Oxford Nanopore Technologies (ONT), UK), with the following major modifications: 1) during tagmentation, 10 ng of AMPure (Beckman, Brea, CA) purified DNA was mixed with 2.5 μl of Fragmentation Mix (FRM); 2) during PCR, 10 μl of tagmented DNA and 2 μl of Rapid Barcode Primer (RLB), 50 μl of LongAmp Taq 2X Master Mix and 38 μl of nuclease-free water were mixed per PCR reaction; 3) The number of PCR cycles was increased to 25 cycles and extension time was reduced to 4 min. After PCR, each sample was purified using 0.6X AMPure beads and quantified using Qubit High sensitivity Kit (Life Technologies, Carlsbad, CA). Equal amounts of each amplicon were pooled together, with a total amount of 50~300 fmol at a volume of 10 μl. Library was loaded to the flow cell (R9.4.1) and sequenced on the MinION platform for an average of 5 h.

Bacterial 16S rRNA gene sequencing and analyses

The hypervariable V4 region of the bacterial 16S rRNA gene was amplified and then sequenced on Illumina MiSeq Platform as per Illumina's recommendations (2) as

previously reported (3). Reads were de-multiplexed onboard the sequencer using standard Illumina software. We performed post-sequencing quality control (QC) using an in-house pipeline developed by the University of Pittsburgh Center for Medicine and the Microbiome (CMM), utilizing open source software including dust, fastx toolkit, and cutadapt (4–6). We applied an in-house mothur pipeline to generate operational taxonomic units (OTU) and ribosomal database project (RDP) classified sequences (7,8). Samples that generated fewer than 100 reads were exclude from further analyses.

We performed permutational multivariate analysis of variance (PERMANOVA) testing to compare microbial community structure between depleted and undepleted samples, and applied the non-metric multidimensional scaling (NMDS) plot using Bray-Curtis dissimilarity index to visualize the clustering of samples, with the R vegan package (9).

Quantitative PCR (qPCR) assays

SYBR GREEN-based qPCR of human Glyceraldehyde 3-phosphate dehydrogenase (GAPDH) gene was performed to detect and quantify human DNA in depleted and undepleted samples on LightCyler System (Roche, Basel, Switzerland). We also applied probe-based qPCR of bacterial 16S rRNA gene (V3-V4 region) to quantify the bacterial load in samples before and after human DNA depletion (10).

Figure S1. Clinical course description and comparisons of microbiologic cultures with sequencing results for all patients enrolled. In each of these 22 plots, we summarize the clinical course over the first 10 days from initiation of mechanical ventilation, the antibiotic usage, chest X-ray findings, clinical pulmonary infection score (CPIS) (11), procalcitonin levels, 16S sequencing and Nanopore sequencing for each case. In bar plots showing 16S sequencing and Nanopore sequencing results, each bar represents a microbe; X-axis represents the relative abundance of each microbe in that community. Bacterial respiratory pathogens are shown in red, typical oral bacteria in gray and fungi in yellow. Clinical pulmonary infection score was calculated based on temperature, white blood cell count, features of tracheal secretions, PaO2/FiO2 ratio, chest X-ray, cultures.

ETA: endotracheal aspirate; BAL: bronchoalveolar lavage; WBC: white blood cell; CPIS: clinical pulmonary infection score; PaO2: partial pressure of arterial oxygen; FiO2: Fractional inhaled concentration of oxygen; CXR: chest X-ray; GS: gram stain; GPC: Gram positive Cocci; GNR: Gram negative Rods; NFR: normal respiratory flora; MSSA: methicillin-susceptible *Staphylococcus aureus*; MRSA: Methicillin-resistant *Staphylococcus aureus*; TMP-SMX: Trimethoprim/sulfamethoxazole.

Culture-positive pneumonia

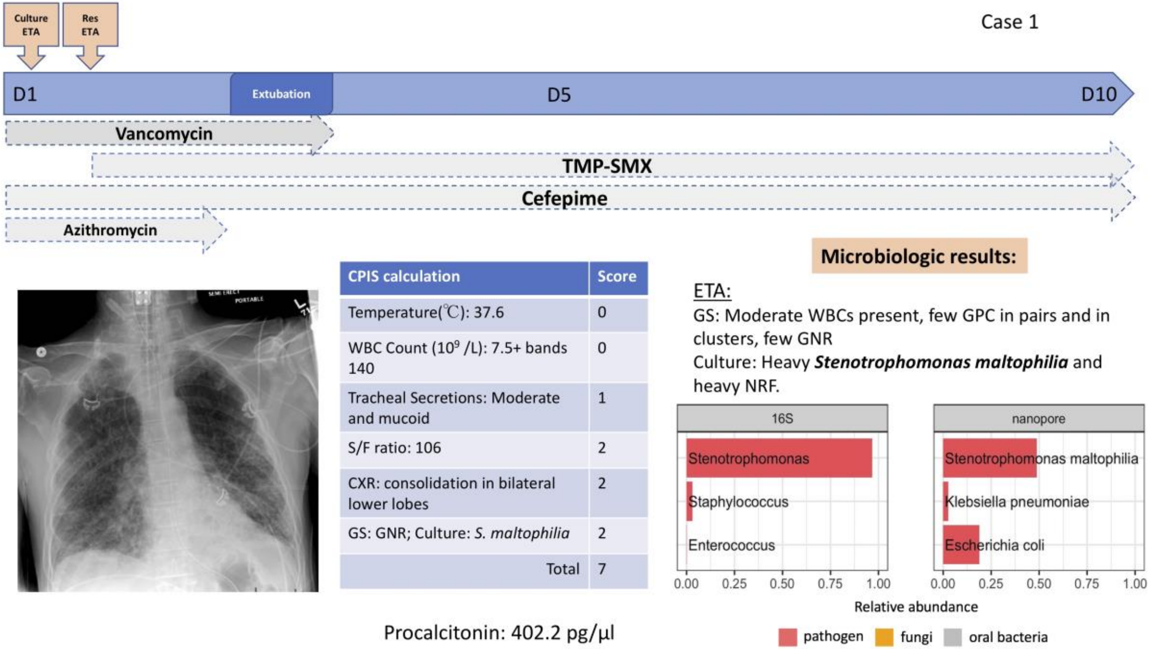

CPIS calculation	Score
Temperature(℃): 37.6	0
WBC Count (10^9 /L): 7.5+ bands 140	0
Tracheal Secretions: Moderate and mucoid	1
S/F ratio: 106	2
CXR: consolidation in bilateral lower lobes	2
GS: GNR; Culture: *S. maltophilia*	2
Total	7

Case 2

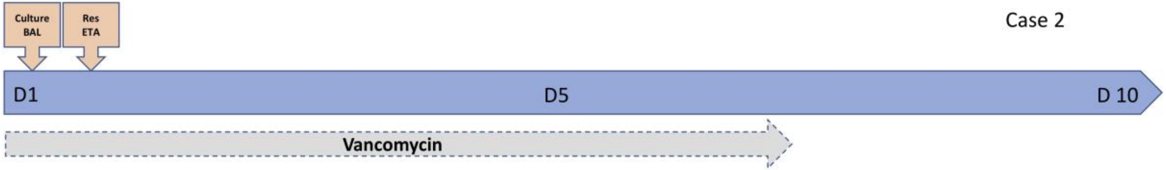

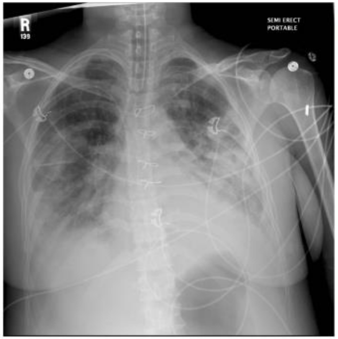

CPIS calculation	Score
Temperature(℃): 34.4	2
WBC Count (10^9/L): 5.7+ bands 114	0
Tracheal Secretions: Moderate and mucoid	1
S/F ratio: 204	2
CXR: Patchy infiltrates	1
Culture: **MRSA**	1
Total	7

Procalcitonin: 4966 pg/µl

Microbiologic results:

BAL:
GS: Moderate WBCs present, no organisms present
Culture: Heavy ***S.aureus (MRSA)***, NRF

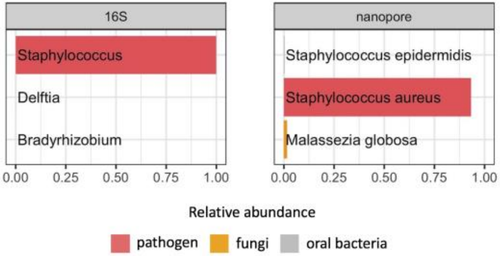

92

Case 3

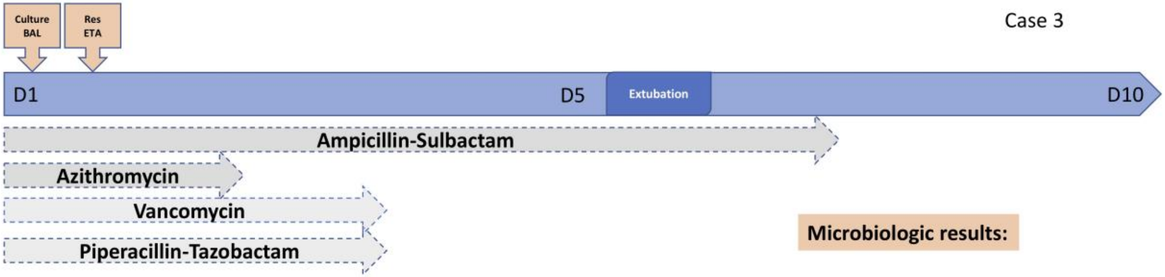

Microbiologic results:

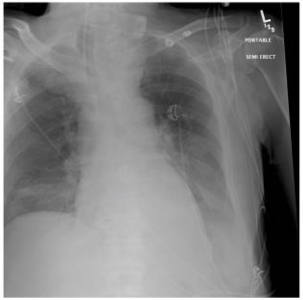

CPIS calculation	Score
Temperature(℃): 35.5	2
WBC Count (10^9/L): 7.4	0
Tracheal Secretions: scant and red	1
S/F ratio: 189	2
CXR: left focal consolidation	2
Culture: ***S.aureus***	1
Total	8

BAL:

GS: Many WBCs present, no organisms seen

Culture: Moderate ***S.aureus (MSSA)***

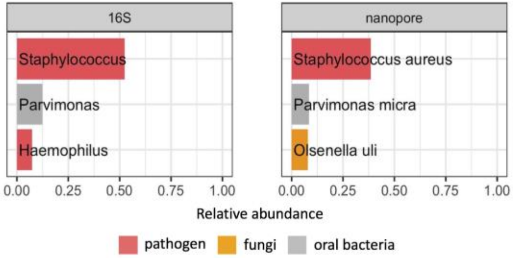

93

Procalcitonin: 1496.43 pg/μl

Case 4

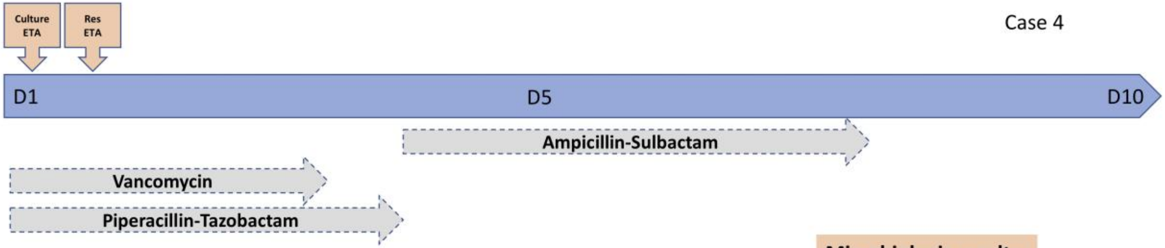

Microbiologic results:

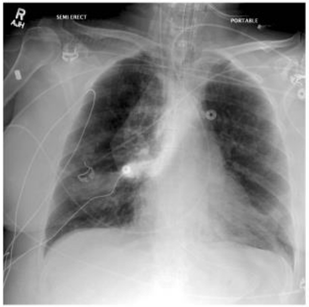

CPIS calculation	Score
Temperature(℃): 38.5	1
WBC Count (10^9/L): 39.8+ 9552 bands	2
Tracheal Secretions: purulent	2
PaO2/FiO2 ratio: 107	2
CXR: Right lower lobe opacities	2
Culture: *E.coli*	1
Total	10

Procalcitonin: 4966 pg/µl

ETA:
GS: Moderate WBCs present, no organisms present
Culture: Light ***E.coli***

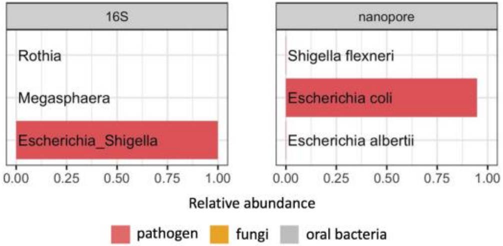

94

Case 5

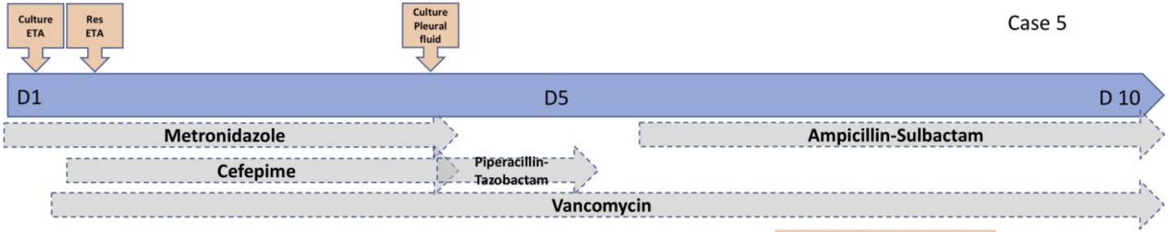

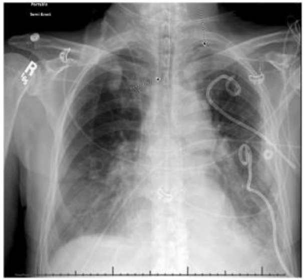

CPIS calculation	Score
Temperature(℃): 38.4	0
WBC Count (10^9/L): 10	0
Tracheal Secretions: scant, tan and thick	1
PaO2/FiO2 ratio: 158	2
CXR: Patchy infrahilar and bibasilar consolidation.	1
GS: GPC; Culture: *S.aureus*	2
Total	6

Procalcitonin: 2783 pg/µl

Microbiologic results:

ETA:

GS: Few WBCs present and few GPC in pairs and in chains, rare yeast
Culture: light ***S.aureus (MSSA)*** and light **NRF.**

Pleural fluid:

GS: Many WBCs present and many GPC
Culture: Heavy ***Strep. Intermedius***, moderate ***Strep. anginosus***, rare ***S.aureus (MSSA)***

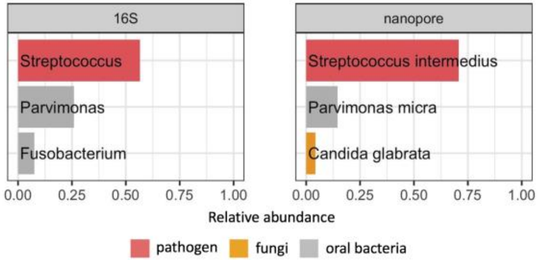

95

Case 6

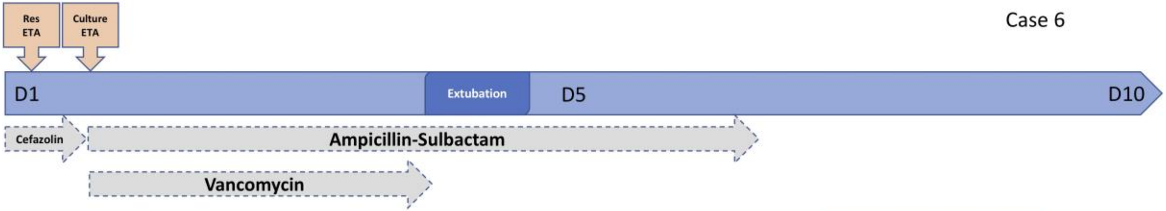

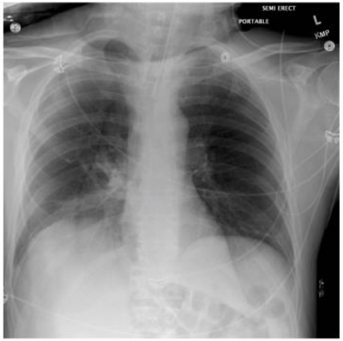

CPIS calculation	Score
Temperature(℃): 37.2	0
WBC Count (10^9/L): 16.8	1
Tracheal Secretions: Purulent	2
PaO2/FiO2 ratio: 220	2
CXR: Right infrahilar infiltrate	2
GS: GPC; Culture: ***S. aureus***	2
Total	9

Procalcitonin: 1049.5 pg/µl

Microbiologic results:

ETA:

GS: Many WBCs present, many Gram Positive Cocci

Culture: Heavy ***S.aureus (MSSA)***

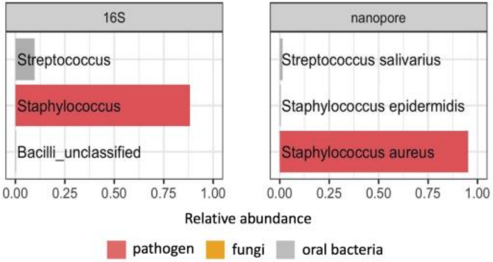

96

Case 7

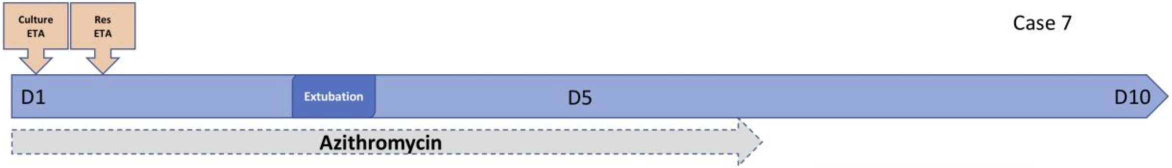

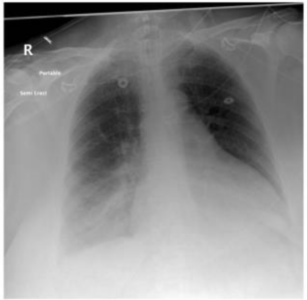

CPIS calculation	Score
Temperature(℃): 35.2	2
WBC Count (10^9/L): 8.3	0
Tracheal Secretions: moderate+ cream	2
PaO2/FiO2 ratio: 108	2
CXR: Pulmonary vascular congestion	0
GS: GNR; Culture: light *Pseudomonas aeruginosa*	2
Total	8

Microbiologic results:

ETA:
GS: Moderate WBCs present, moderate GPC, few GNR
Culture: Light *Pseudomonas aeruginosa*, light NRF

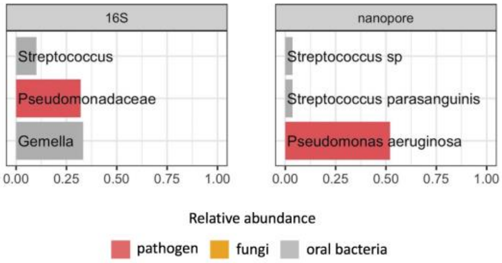

Procalcitonin: 75.19 pg/µl

97

Case 8

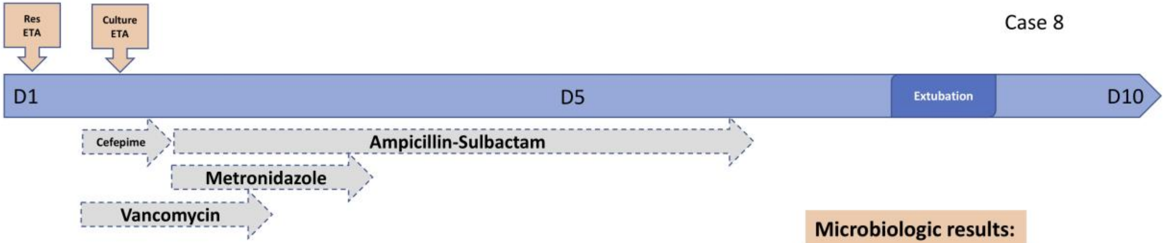

Microbiologic results:

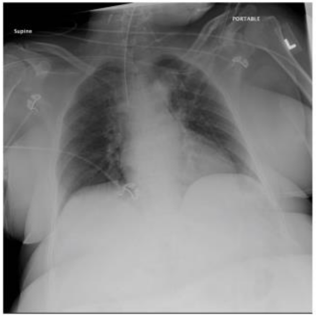

CPIS calculation	Score
Temperature(℃): 35.9	2
WBC Count (10^9/L): 12.4	1
Tracheal Secretions: purulent	2
S/F ratio: 134	2
CXR: Left apical infiltrate	2
GS: yeast; Culture: *S. agalactiae*	1
Total	10

Procalcitonin : 2895.67 pg/µl

ETA:

Gram Stain: Moderate WBCs present, rare Yeast

Culture: Light Group B Streptococci (*S. agalactiae*), NRF

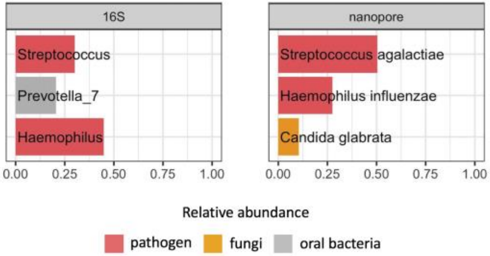

98

Case 9

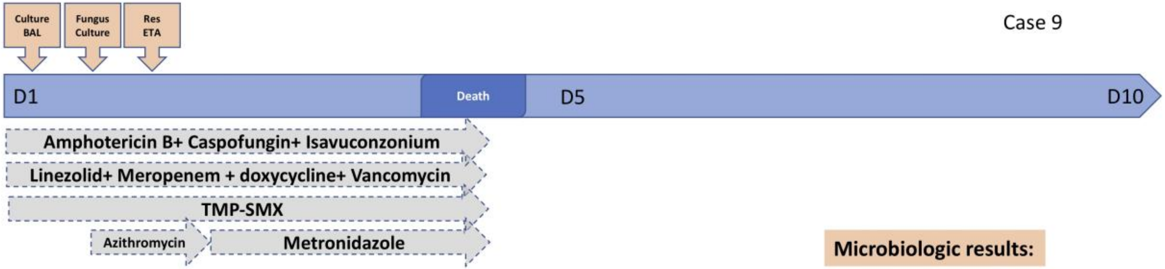

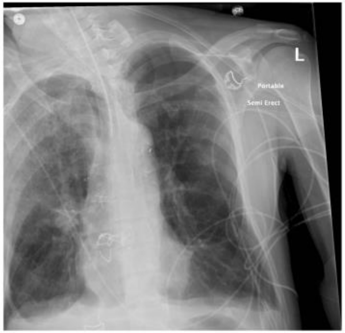

CPIS calculation	Score
Temperature(℃): 35.3	2
WBC Count (10^9/L): 20.9	1
Tracheal Secretions: Purulent	2
PaO2/FiO2 ratio: 442	0
CXR: Right upper lobe consolidation	2
Culture: Yeast+ *Aspergillus*	2
Total	9

Procalcitonin: 4330.13pg/μl

Microbiologic results:

ETA:
GS: Many WBCs present, few Yeast
Culture: *Aspergillus fumigatus*, yeast, not cryptococcus species

Fungal detection not available by 16S

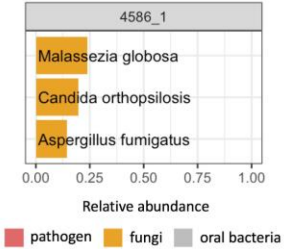

99

Culture-negative pneumonia

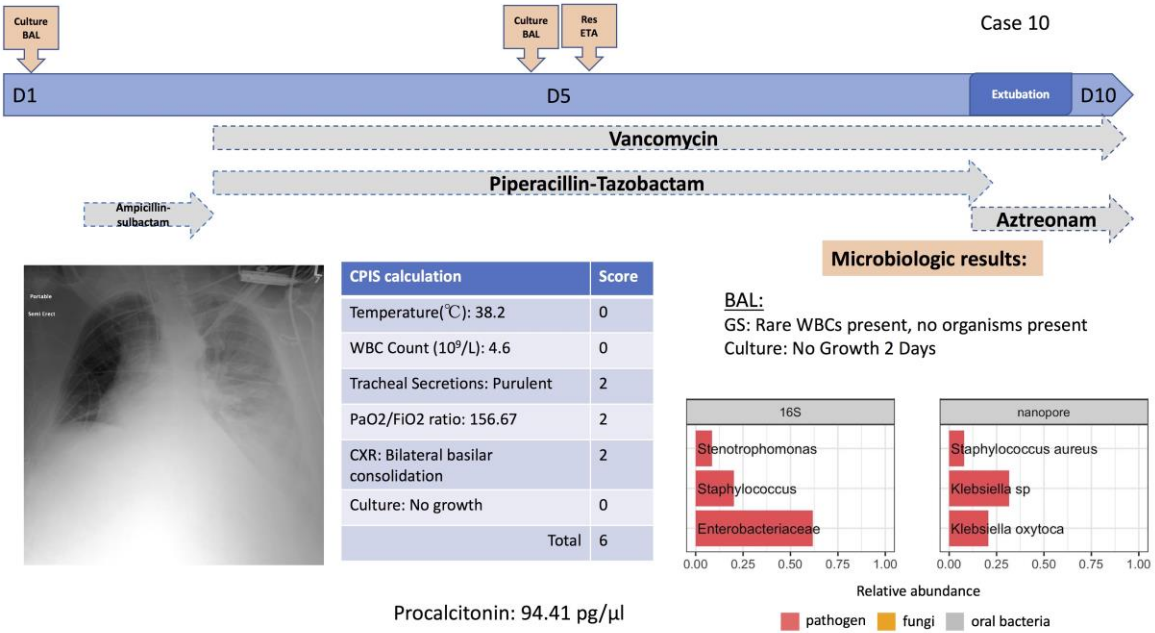

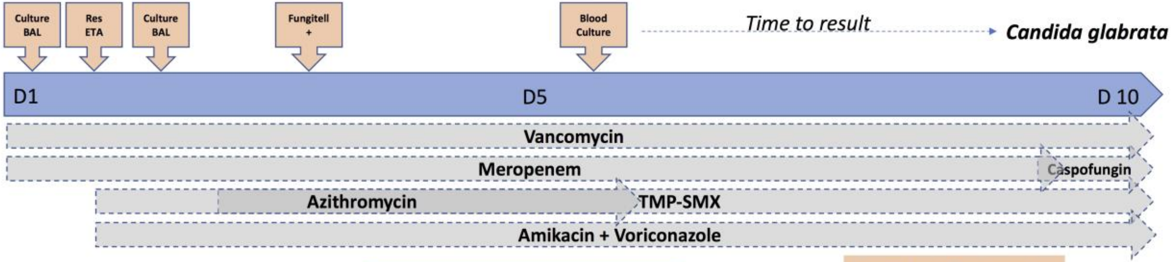

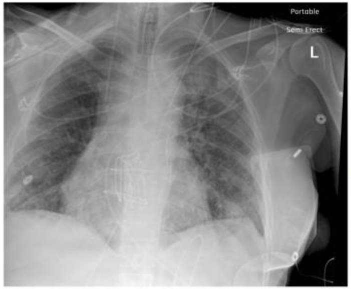

CPIS calculation	Score
Temperature(℃): 38.5	1
WBC Count (10^9/L): 3.5	1
Tracheal Secretions: Purulent	2
S/F ratio: 136	2
CXR: Patchy airspace opacity bilaterally	1
GS: Yeast; Culture: Yeast	2
Total	9

Microbiologic results:

BAL:
GS: Many WBCs present, few Yeast
Culture: Yeast, not cryptococcus species, NRF

Fungal detection not available by 16S

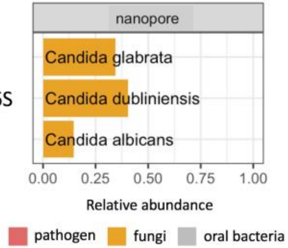

102 Case 11

Procalcitonin: 4866 pg/µl Fungitell 283 pg/µl

Case 12

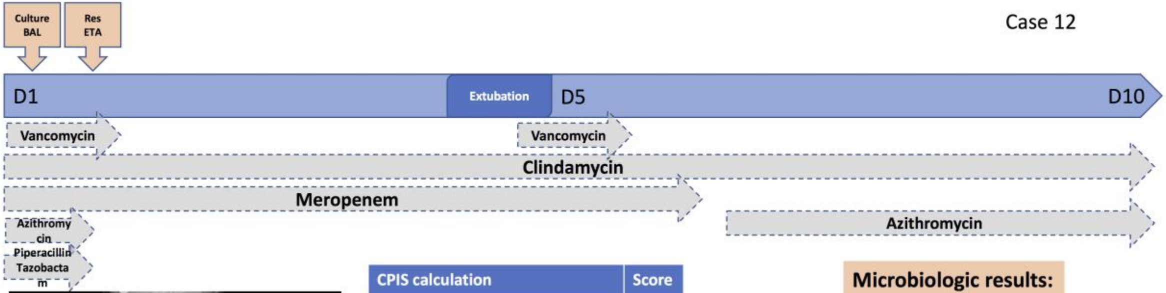

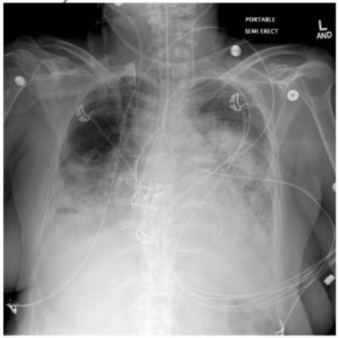

CPIS calculation	Score
Temperature(℃): 37.3	0
WBC Count (10^9/L): 2.6+ bands 208	1
Tracheal Secretions: purulent	2
PaO2/FiO2 ratio: 206	0
CXR: Bilateral consolidation, left greater than right	2
Culture: no growth	0
Total	5

Procalcitonin : 16,000 pg/ul

Microbiologic results:

BAL:

GS: Moderate WBCs present, no organisms seen

Culture: No Growth 2 Days

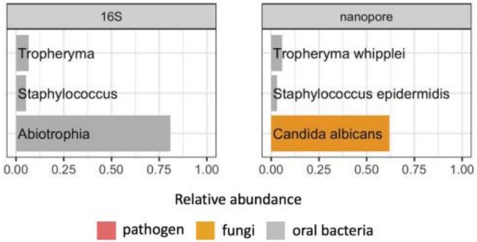

103

Case 13

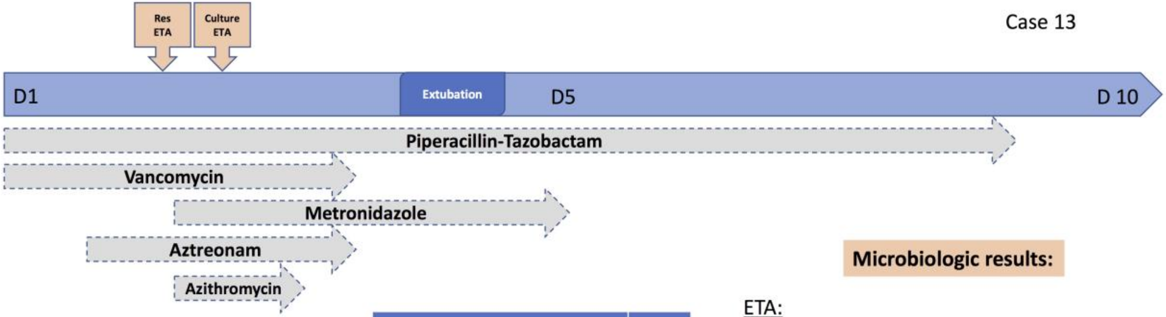

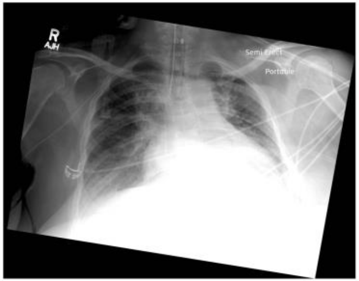

CPIS calculation	Score
Temperature(℃): 37.4	0
WBC Count (10^9/L): 8.3	0
Tracheal Secretions: scant and clear	1
PaO2/FiO2 ratio: 112	2
CXR: Bilateral patchy opacities	1
Culture: NRF	0
Total	4

Procalcitonin: 30.55 pg/µl

Microbiologic results:

ETA:

GS: Many WBCs present, No organisms seen

Culture: Rare NRF

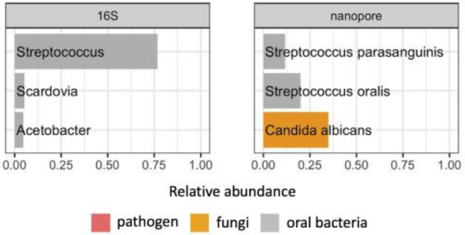

104

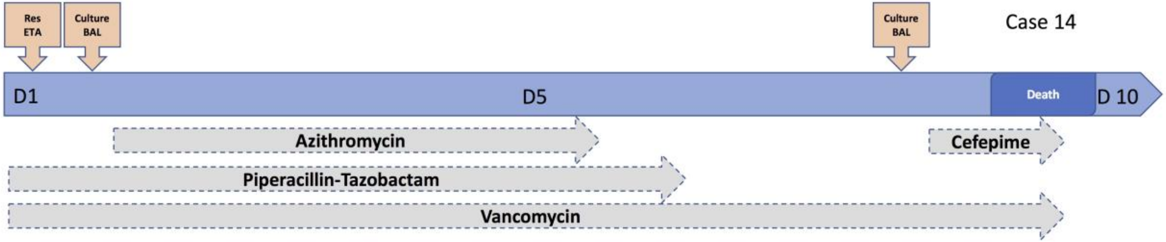

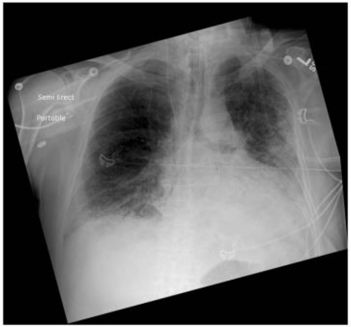

CPIS calculation	Score
Temperature(℃): 37.2	0
WBC Count (10^9/L): 39.6+ bands 1584	2
Tracheal Secretions: scant and mucoid	1
PaO2/FiO2 ratio: 86	2
CXR: Left lung and bibasilar opacities.	2
Culture: NRF	0
Total	7

Procalcitonin: 4965.08 pg/μl

Microbiologic results:

BAL:

GS: Few WBCs present, no organisms seen

Culture: NRF

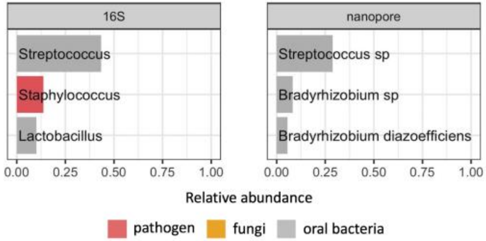

Controls – Congestive Heart Failure

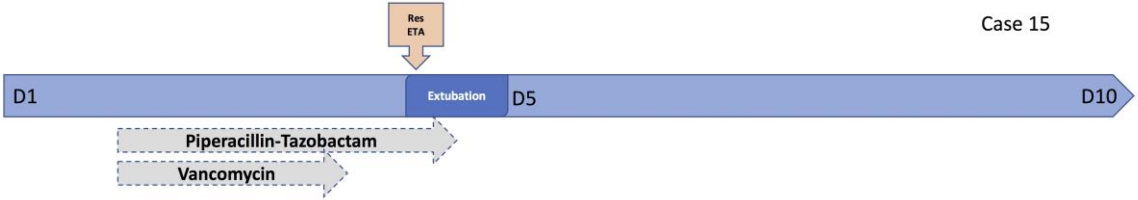

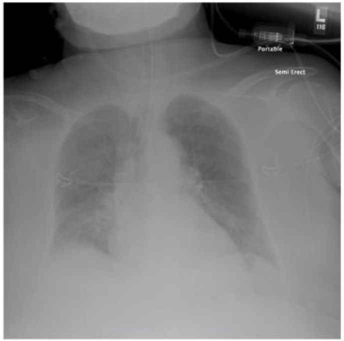

CPIS calculation	Score
Temperature(℃): 37.9	0
WBC Count (10^9/L): 17.7	1
Tracheal Secretions: Purulent	2
PaO2/FiO2 ratio: 311.6	0
Pulmonary Radiography: Bilateral pulmonary infiltrates	1
Culture of tracheal aspirate: not available	0
Total	4

Procalcitonin : 2783 pg/µl
BNP: 379 pg/ml

Microbiologic results:

Cultures:
Not available

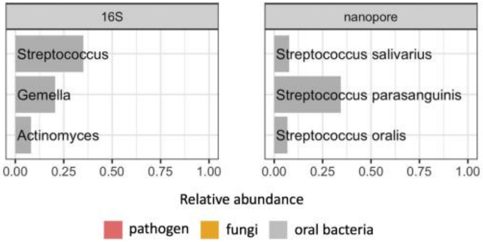

Case 16

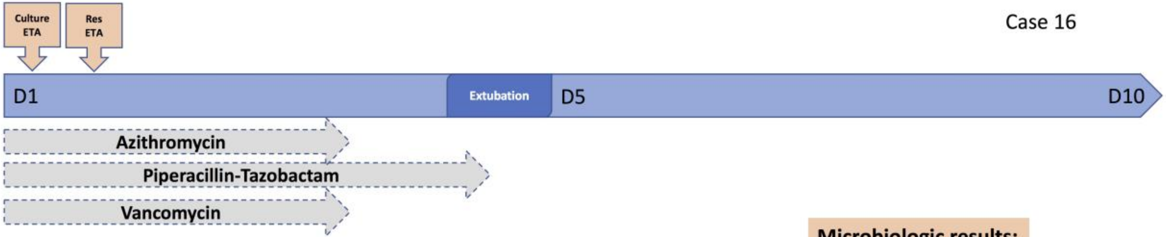

Microbiologic results:

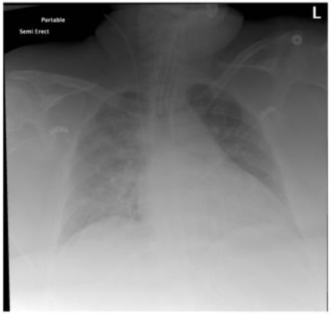

CPIS calculation	Score
Temperature(℃): 35.8	2
WBC Count (10^9/L): 5.3	0
Tracheal Secretions: purulent	2
PaO2/FiO2 ratio: 225	2
CXR: Bilateral pulmonary infiltrates	1
Culture: rare NRF	0
Total	7

ETA:
GS: Moderate WBCs present, no organisms present
Culture: NRF

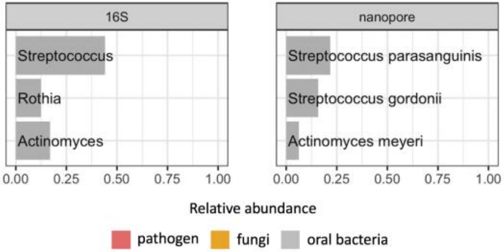

Procalcitonin : 2783 pg/µl
BNP: 184 pg/ml

108

Case 17

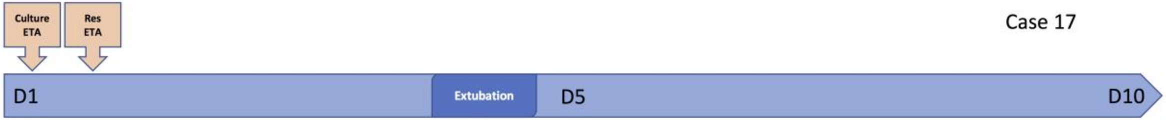

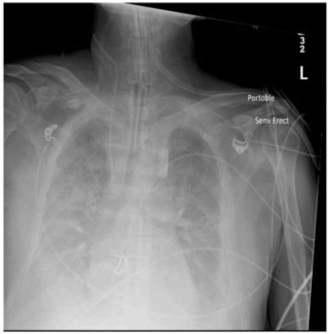

CPIS calculation	Score
Temperature(℃): 35.1	2
WBC Count (10^9/L): 3.7	1
Tracheal Secretions: purulent	2
S/F ratio: 273	0
CXR: Bilateral pulmonary infiltrates	1
Culture of tracheal aspirate: moderate normal oral flora	0
Total	6

Procalcitonin : 393.04 pg/μl
BNP: 1889 pg/ml

Microbiologic results:

ETA:
GS: Many WBCs present, few GPC in pairs and in clusters
Culture: Moderate NRF

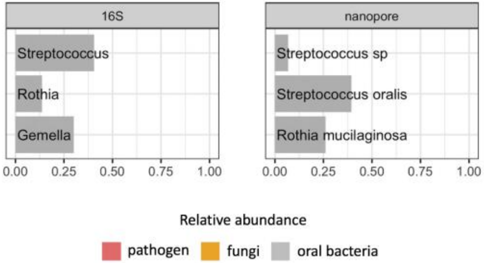

109

Controls – Airway Protection

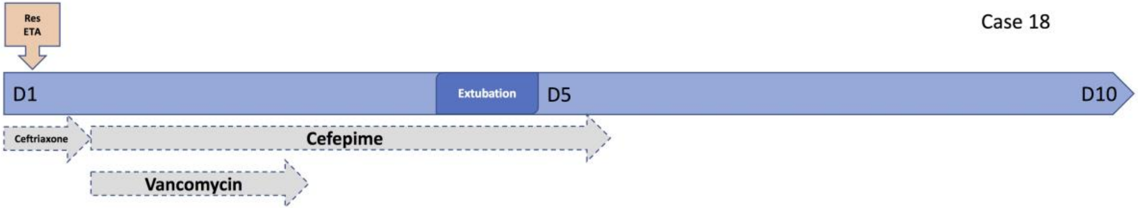

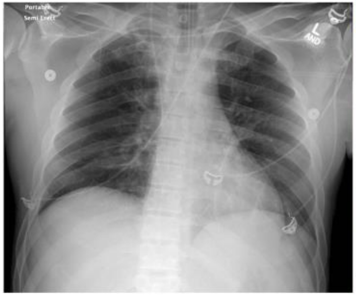

CPIS calculation	Score
Temperature(℃): 36	2
WBC Count (10^9/L): 16.7	1
Tracheal Secretions: scant and white	2
PaO2/FiO2 ratio: 453.33	0
CXR: No abnormality	0
Culture of tracheal aspirate: not available	0
Total	5

Procalcitonin : 313.92 pg/μl

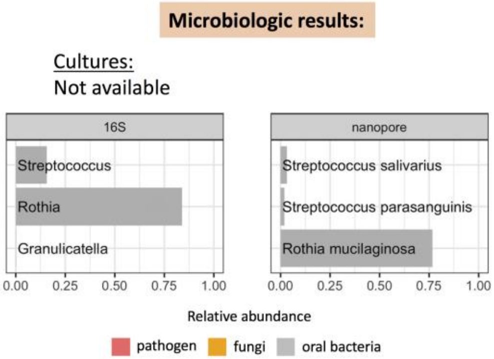

Case 19

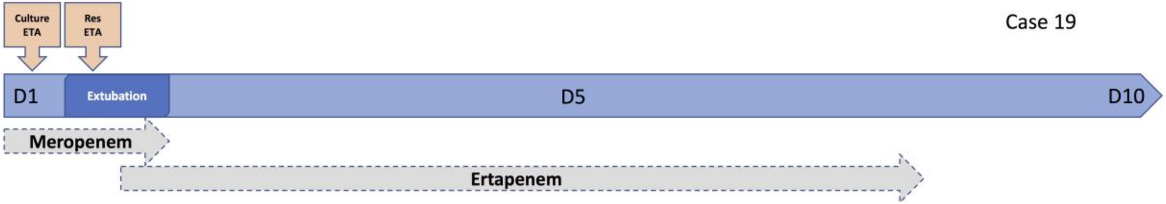

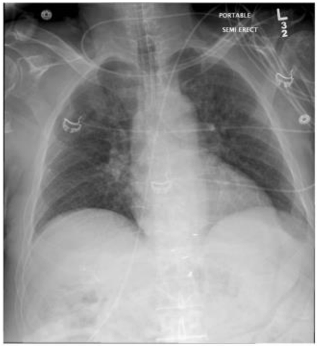

CPIS calculation	Score
Temperature(℃): 35.4	2
WBC Count (10^9/L): 4.3	0
Tracheal Secretions: Scant and Clear	1
PaO2/FiO2: >273	0
CXR: No abnormality	0
Culture: rare NRF	0
Total	3

Procalcitonin : 247.75 pg/μl

Microbiologic results:

ETA:
GS: WBCs present, rare Epithelial cells present, no organisms present
Culture: Rare NRF

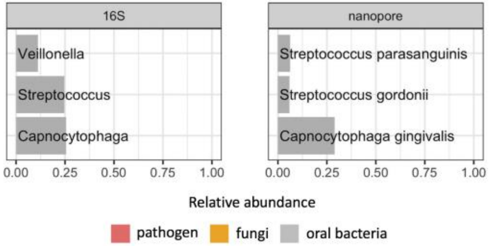

112

Case 20

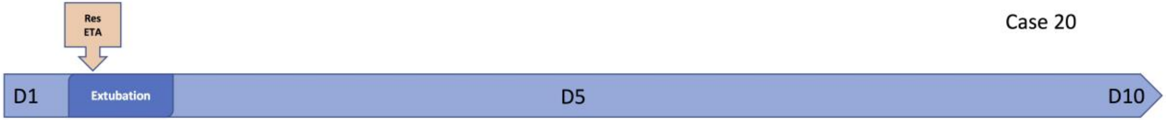

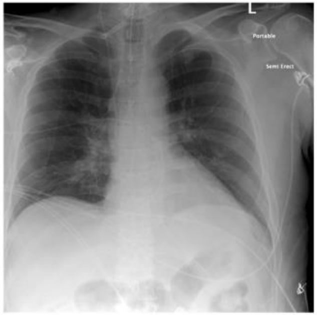

CPIS calculation	Score
Temperature(℃): 35.5	2
WBC Count (10^9/L): 10.7	0
Tracheal Secretions: scant and mucoid	1
PaO2/FiO2 ratio: 237	2
CXR: Unremarkable	0
Culture: not available	0
Total	5

Procalcitonin : 251.23 pg/µl

Microbiologic results:

Cultures:
Not available

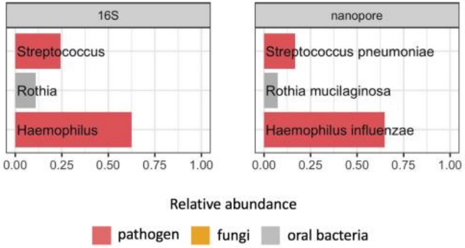

113

Case 21

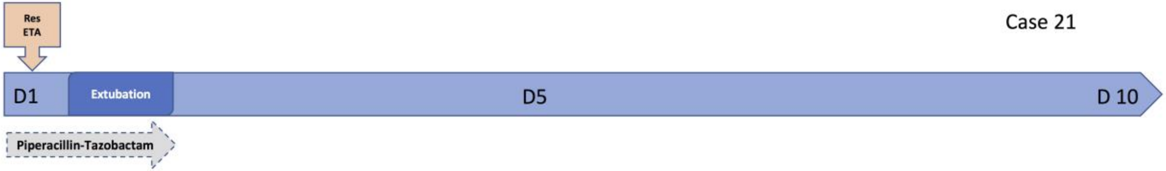

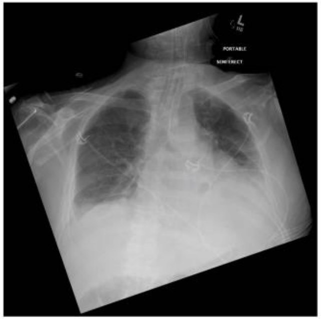

CPIS calculation	Score
Temperature(℃): 35.9	2
WBC Count (10^9/L): 4.5	0
Tracheal Secretions: scant and clear	1
PaO2/FiO2 ratio: 176	2
CXR: Unremarkable	0
Culture: not available	0
Total	5

Procalcitonin: 1114.44 pg/µl

Microbiologic results:

Cultures:
Not available

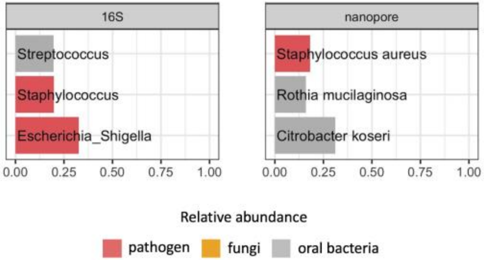

114

Case 22

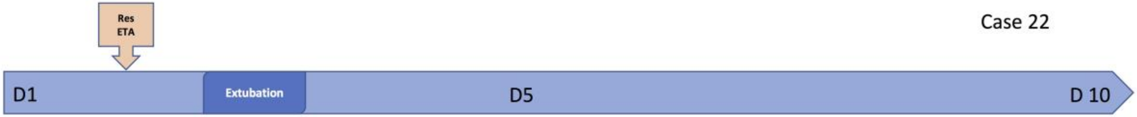

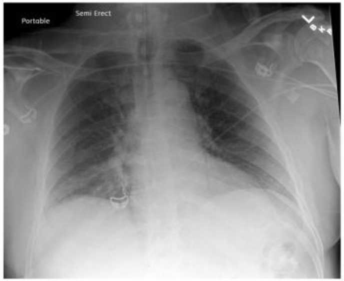

CPIS calculation	Score
Temperature(℃): 36.3	0
WBC Count (10^9/L): 6.3	0
Tracheal Secretions: Purulent	2
PaO2/FiO2 ratio: 236	2
CXR: Unremarkable	0
Culture: not available	0
Total	4

Procalcitonin: 56.54 pg/μl

Microbiologic results:

Cultures:
Not available

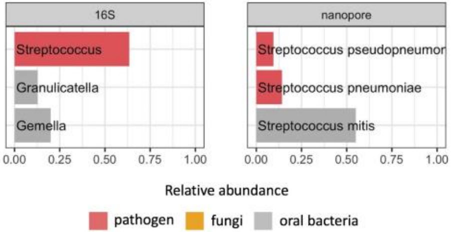

Figure S2. Comparison of clinical pulmonary infection scores (CPIS) and plasma procalcitonin between the three clinical groups. (A) Patients with culture-positive pneumonia had significantly higher CPIS compared to controls (Wilcoxon test, p=0.0014). (B) Patients with culture-positive pneumonia had a trend for higher Procalcitonin compared to controls.

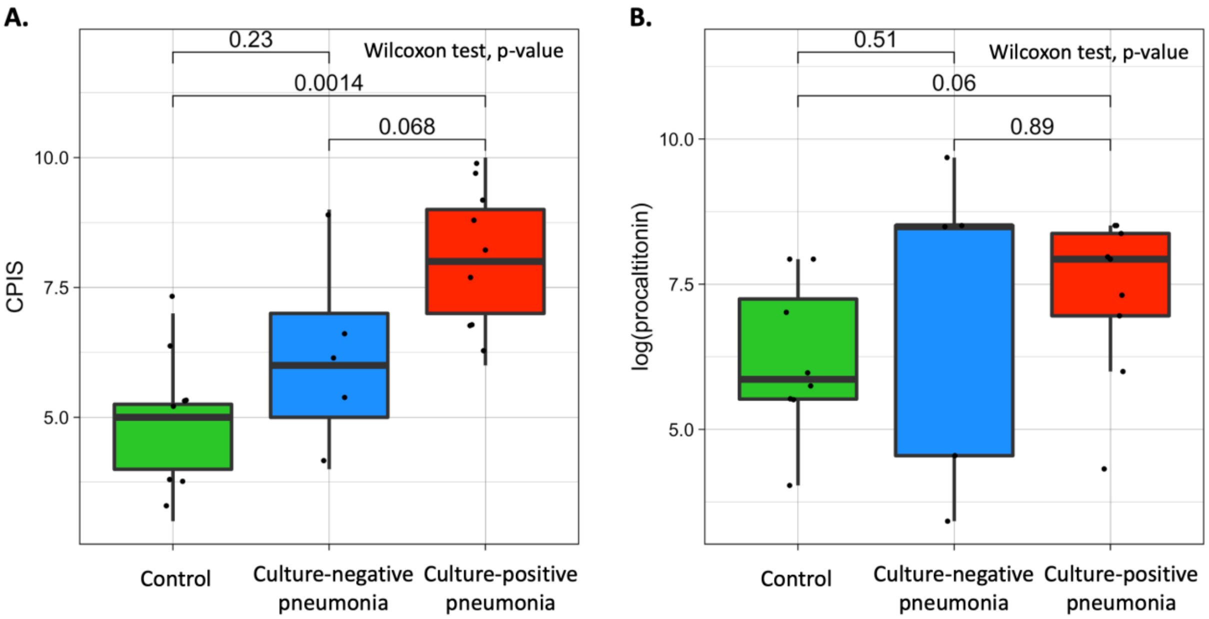

Figure S3. Human DNA depletion resulted in reduction of human DNA by 1260-fold without changing bacterial DNA levels. (A) We quantified bacterial DNA level by performing qPCR of bacteria 16S rRNA gene. There was no significant difference in qPCR cycle threshold (Ct) value between depleted and undepleted samples (paired sample t-test, p-value=0.8315). (B) We quantified human DNA load in depleted and undepleted ETAs by performing qPCR of human GAPDH gene. Compared to undepleted samples, the qPCR Ct value of depleted samples was significantly increased after depletion (paired samples t-test, p-value=0.0028)**.** The Ct value increased by an average of 10.3 cycles after saponin depletion, corresponding to 1260-fold deduction of human DNA.

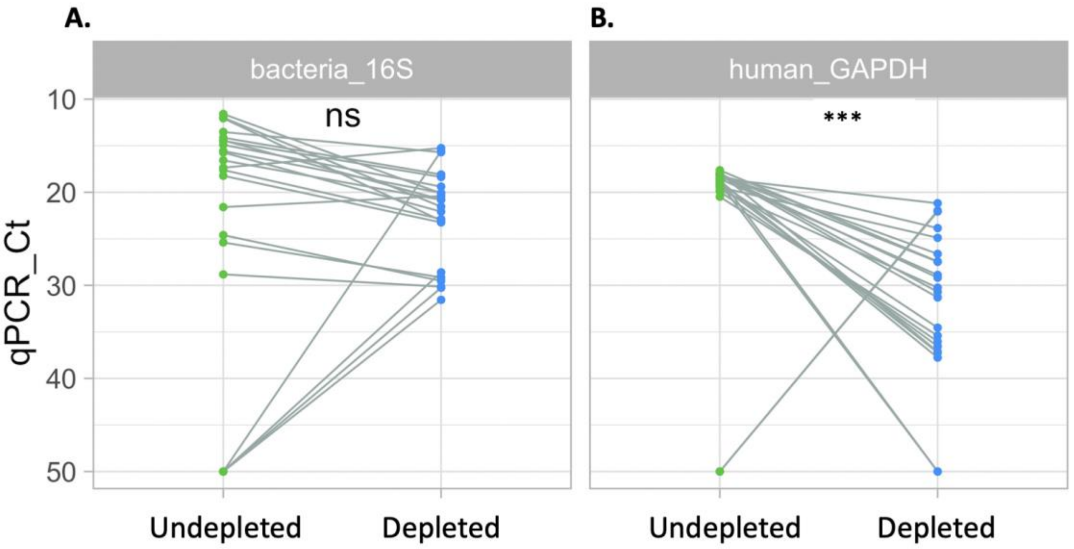

Table S1. Nanopore sequencing effectively reproduces the expected composition of a mock microbial community. To examine the quality and reproducibility of Nanopore metagenomic sequencing, we sequenced a reference mock community from Zymo (Irvine, CA) in duplicates. The Microbial Community Standard mimics a mixed microbial community of well-defined composition (shown below). Nanopore sequencing successfully identified these microbes and the composition of the sequenced communities closely matched the expected composition based on the manufacturer's data. The only notable discrepancies between observed and expected microbial composition had to do with lower than expected reads of *Pseudomonas aeruginosa*, and identification of *Enterococcus faecium* which should not have been present in the mock community, but likely represented the closely related organism *Enterococcus faecalis*.

Species	Expected Composition of Genomic DNA (%)	Sample 1 Nanopore reads number (%)	Sample 2 Nanopore reads number (%)
Escherichia coli	12.0	18,788 (14.5)	17,942 (16.0)
Salmonella enterica	12.0	15,059 (11.6)	12,328 (11.0)
Lactobacillus fermentum	12.0	12,230 (9.5)	10,976 (9.8)
Enterococcus faecalis	12.0	13,915 (10.8)	10,379 (9.2)
Staphylococcus aureus	12.0	18,160 (14.0)	17,298 (15.4)
Listeria monocytogenes	12.0	19,628 (15.2)	16,352 (14.6)
Bacillus subtilis	12.0	18,329 (14.2)	15,178 (13.5)
Pseudomonas aeruginosa	12.0	7,363 (5.7)	6,468 (5.8)
Saccharomyces cerevisiae	2.0	1,970 (1.5)	1,515 (1.3)

Cryptococcus neoformans	2.0	2,992 (2.3)	3,022 (2.7)
Homo sapiens	0.0	495 (0.4)	428 (0.4)
Enterococcus faecium	0.0	398 (0.3)	485 (0.4)

Figure S4. Comparisons of lung microbial communities between culture-positive and culture-negative samples by Nanopore sequencing (A) Culture-positive samples had a trend for lower alpha diversity of lung microbiome compared to culture-negative samples (including both cases of culture-negative pneumonia and controls) by Shannon index. (B) On NMDS plot, there was significant difference in overall lung microbial community structure between culture-positive and negative samples (PERMANOVA for Bray-Curtis dissimilarity index, p=0.039, R^2=0.066).

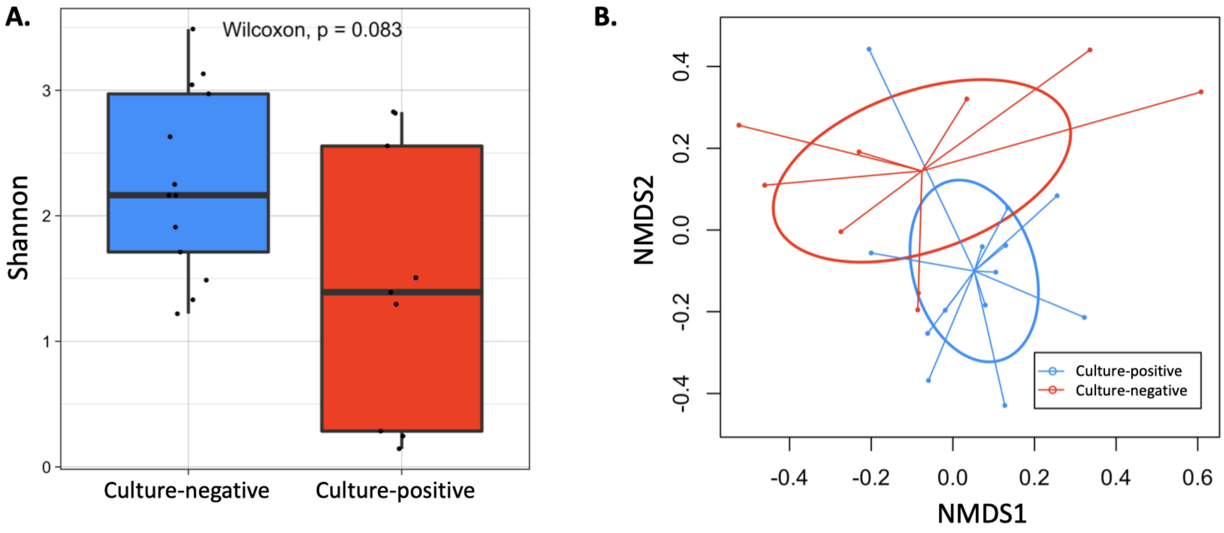

References:

1. Charalampous T, Richardson H, Kay GL, Baldan R, Jeanes C, Rae D, et al. Rapid Diagnosis of Lower Respiratory Infection using Nanopore-based Clinical Metagenomics. BioRxiv. 2018 Aug 9;
2. Caporaso JG, Lauber CL, Walters WA, Berg-Lyons D, Huntley J, Fierer N, et al. Ultra-high-throughput microbial community analysis on the Illumina HiSeq and MiSeq platforms. ISME J. 2012 Aug;6(8):1621–4.
3. Kitsios GD, Fitch A, Manatakis DV, Rapport SF, Li K, Qin S, et al. Respiratory microbiome profiling for etiologic diagnosis of pneumonia in mechanically ventilated patients. Front Microbiol. 2018 Jul 10;9:1413.
4. Morgulis A, Gertz EM, Schäffer AA, Agarwala R. A fast and symmetric DUST implementation to mask low-complexity DNA sequences. J Comput Biol. 2006 Jun;13(5):1028–40.
5. Blankenberg D, Gordon A, Von Kuster G, Coraor N, Taylor J, Nekrutenko A, et al. Manipulation of FASTQ data with Galaxy. Bioinformatics. 2010 Jul 15;26(14):1783–5.
6. Martin M. Cutadapt removes adapter sequences from high-throughput sequencing reads. EMBnet j. 2011 May 2;17(1):10.
7. Schloss PD, Westcott SL, Ryabin T, Hall JR, Hartmann M, Hollister EB, et al. Introducing mothur: open-source, platform-independent, community-supported software for describing and comparing microbial communities. Appl Environ Microbiol. 2009 Dec;75(23):7537–41.
8. Wang Q, Garrity GM, Tiedje JM, Cole JR. Naive Bayesian classifier for rapid assignment of rRNA sequences into the new bacterial taxonomy. Appl Environ Microbiol. 2007 Aug;73(16):5261–7.
9. Dixon P. VEGAN, a package of R functions for community ecology. Journal of Vegetation Science. 2003 Dec;14(6):927–30.
10. Liu CM, Aziz M, Kachur S, Hsueh P-R, Huang Y-T, Keim P, et al. BactQuant: an enhanced broad-coverage bacterial quantitative real-time PCR assay. BMC Microbiol. 2012 Apr 17;12:56.
11. Zilberberg MD, Shorr AF. Ventilator-associated pneumonia: the clinical pulmonary infection score as a surrogate for diagnostics and outcome. Clin Infect Dis. 2010 Aug 1;51 Suppl 1:S131-5.
